# EEG Functional Connectivity Associated With Antidepressant Response to Transcutaneous Electrical Cranial‐Auricular Acupoint Stimulation

**DOI:** 10.1002/cns.70920

**Published:** 2026-07-01

**Authors:** Yanan Zhao, Zhouzhi Yin, Ying Zhang, Xiaoqiu Wang, Chengyong Liu, Wenzhong Wu, Peijing Rong

**Affiliations:** ^1^ Institute of Acupuncture and Moxibustion China Academy of Chinese Medical Sciences Beijing China; ^2^ Nanjing University of Chinese Medicine Nanjing Jiangsu China; ^3^ Department of Preventive Medicine Affiliated Hospital of Nanjing University of Chinese Medicine Nanjing Jiangsu China; ^4^ Department of Acupuncture and Moxibustion and Rehabilitation Affiliated Hospital of Nanjing University of Chinese Medicine Nanjing Jiangsu China; ^5^ Institute of Basic Clinical Medicine of Chinese Medicine China Academy of Chinese Medical Sciences Beijing China

**Keywords:** antidepressant prediction, biomarker, electroencephalography, major depressive disorder, transcutaneous electrical cranial‐auricular acupoint stimulation

## Abstract

**Aims:**

Transcutaneous electrical cranial‐auricular acupoint stimulation (TECAS) is an emerging neuromodulation therapy that integrates principles of acupuncture with noninvasive electrical stimulation. While TECAS has demonstrated efficacy in treating major depressive disorder (MDD), patient responses vary considerably, and the neurophysiological mechanisms remain poorly understood. This clinical study aimed to investigate electroencephalography (EEG)‐based functional connectivity (FC) as a potential predictor of clinical response to TECAS in patients with MDD.

**Methods:**

Resting‐state EEG was recorded from 50 MDD patients before and after 8 weeks of TECAS treatment, and from 37 healthy controls (HCs). We compared baseline FC between MDD patients and HCs, pre‐to‐posttreatment changes in patients, and baseline features between treatment responders and nonresponders. Correlations between FC features and clinical symptom improvements were also assessed.

**Results:**

After treatment, frontoparietal θ‐band connectivity strength was significantly reduced in MDD. Crucially, pretreatment frontoparietal δ‐band connectivity was negatively correlated with posttreatment reductions in depression and anxiety scores. Furthermore, responders demonstrated significantly higher pretreatment β‐band FC than nonresponders.

**Conclusion:**

Our findings indicate that TECAS modulates frontoparietal θ connectivity in MDD. More importantly, pretreatment δ‐ and β‐band FC patterns are associated with therapeutic outcome. These features may serve as potential biomarkers for predicting response to TECAS, facilitating the development of personalized therapeutic strategies for MDD.

## Graphical abstract

(A) Fifty MDD patients underwent an 8‐week TECAS treatment and were then classified into responders and nonresponders according to their clinical outcomes. (B) Resting‐state EEG was acquired from all participants and subsequently preprocessed. (C) Functional connectivity analysis revealed that TECAS modulates frontoparietal θ‐band connectivity in MDD, and pretreatment δ‐ and β‐band FC patterns are associated with therapeutic outcome.

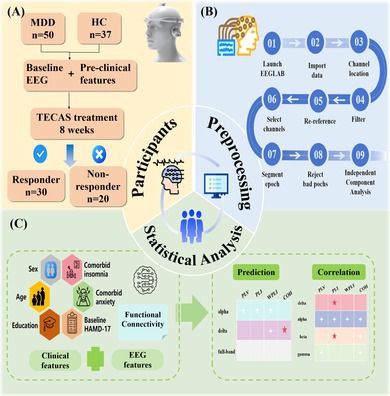

**Abbreviations**
COH: coherence
DSM‐5: Diagnostic and Statistical Manual of Mental Disorders, Fifth Edition
ECT: electroconvulsive therapy
EEG: electroencephalography
FC: functional connectivity
FIR: finite impulse response
fMRI: functional magnetic resonance imaging
FPN: frontoparietal network
HAMA‐14: 14‐item Hamilton Anxiety Scale
HAMD‐17: 17‐item Hamilton Depression Rating Scale
HCs: healthy controls
ICA: independent component analysis
ICD‐10: International Classification of Diseases, 10th Revision
IQR: interquartile range
MDD: major depressive disorder
NBS: Network‐Based Statistic
NTS: nucleus tractus solitarius
PLI: phase lag index
PLV: phase‐locking value
rTMS: repetitive transcranial magnetic stimulation
SCID: Structured Clinical Interview for ICD‐10
SCID‐5: Structured Clinical Interview for DSM‐5
taVNS: transcutaneous auricular vagus nerve stimulation
TECAS: transcutaneous electrical cranial‐auricular acupoint stimulation
WPLI: weighted phase lag index

## 1 Introduction

Major depressive disorder (MDD) constitutes a substantial global public health burden, affecting hundreds of millions of individuals and ranking among the leading causes of disability worldwide [1, 2]. Although pharmacological treatments remain the cornerstone of clinical management, their effectiveness is frequently constrained by heterogeneous treatment responses and considerable adverse effects [3, 4]. This clinical reality highlights a pressing need for safe, effective, and mechanistically distinct non‐pharmacological therapeutic alternatives [5, 6].

Acupuncture has increasingly attracted attention as a potential intervention for MDD, with accumulating evidence suggesting that its antidepressant effects may be mediated through enhanced hippocampal neuroplasticity and attenuation of neuroinflammatory processes [7]. Based on the neuroanatomical and functional relevance of key acupoints—such as Baihui (GV20) and Yintang (GV29) on the scalp, as well as the Heart and Liver points on the auricle—we developed transcutaneous electrical cranial‐auricular acupoint stimulation (TECAS). This novel modality integrates principles of traditional acupuncture with contemporary neuromodulation technology, enabling precise control of stimulation parameters while maintaining noninvasiveness and feasibility for home‐based use. Previous clinical trials have demonstrated that TECAS achieves an antidepressant response rate of 66.4%, comparable to that of escitalopram [8], and confers additional benefits on comorbid symptoms, including insomnia [9]. Furthermore, neuroimaging studies indicate that TECAS modulates functional connectivity (FC) within core brain networks implicated in MDD, such as the default mode network and frontoparietal circuits [10, 11, 12]. Nevertheless, its impact on neural electrophysiological dynamics—particularly electroencephalography (EEG)‐based FC—has yet to be systematically investigated.

EEG offers a unique window into large‐scale brain network dynamics owing to its millisecond‐level temporal resolution. Neural oscillations orchestrate coordinated activity across distributed cortical regions and constitute a fundamental mechanism underlying functional brain networks. Converging evidence suggests that the pathophysiology of MDD is characterized by disturbances in large‐scale network organization [13, 14, 15, 16], commonly reflected in altered cortical excitation–inhibition balance and maladaptive hyperactivity patterns [17]. Consistent EEG findings in MDD include reduced γ‐band activity, increased θ‐band connectivity, and disrupted interhemispheric communication [18, 19], all of which have been linked to core depressive symptoms such as cognitive impairment and maladaptive rumination [20]. Importantly, EEG‐derived FC measures have shown promise as predictive biomarkers for treatment response across multiple interventions, including antidepressant pharmacotherapy, electroconvulsive therapy (ECT), and repetitive transcranial magnetic stimulation (rTMS) [21, 22, 23, 24, 25, 26, 27, 28].

Despite its demonstrated therapeutic potential, substantial inter‐individual variability in response to TECAS persists. The present study aims to identify EEG‐based FC markers capable of predicting antidepressant response to TECAS in patients with MDD. By characterizing frequency‐specific network features that distinguish responders from nonresponders and examining their associations with clinical symptom improvement, we aim to explore potential neurophysiological biomarkers that could inform individualized TECAS treatment.

## 2 Materials and Methods

### 2.1 Study Design

This was a prospective, single‐arm interventional study. All patients diagnosed with MDD received TECAS for 8 consecutive weeks. Depression severity was assessed using the 17‐item Hamilton Depression Rating Scale (HAMD‐17) [29] at baseline and at week 8. Anxiety symptoms were evaluated using the 14‐item Hamilton Anxiety Scale (HAMA‐14) [30]. Resting‐state EEG data were collected at baseline and after the 8‐week intervention in patients with MDD, and at baseline only in healthy controls (HCs). Clinical response was predefined as a ≥ 50% reduction in HAMD‐17 score from baseline to week 8.

### 2.2 Participants

#### 2.2.1 Patients With MDD

First‐episode, treatment‐naïve outpatients with MDD were recruited from the Beijing First Hospital of Integrated Chinese and Western Medicine. The diagnosis of MDD was established by experienced psychiatrists using the Chinese version of the Modified Structured Clinical Interview for ICD‐10 (SCID). Inclusion criteria were as follows [31]: (1) right‐handedness; (2) age between 18 and 65 years; (3) HAMD‐17 total score > 7 and ≤ 24; and (4) all responses rated as “No” or scores < 3 on the Columbia–Suicide Severity Rating Scale (C‐SSRS).

Exclusion criteria included a history of neurological disorders, other major psychiatric disorders, severe systemic diseases, significant physical trauma, pregnancy, or any condition that could interfere with EEG recording or TECAS treatment. Patient safety was monitored throughout the study period.

#### 2.2.2 HCs

HCs were recruited from the local community. The absence of psychiatric disorders was confirmed using the Structured Clinical Interview for DSM‐5 (SCID‐5). Additional inclusion criteria required HAMD‐17 and HAMA‐14 scores below 7. All HCs were right‐handed and free of major medical conditions. Exclusion criteria included a history of substance abuse, brain injury, or inability to maintain a regular daily schedule. The same EEG acquisition protocol and pre‐recording restrictions were applied to HCs as to patients with MDD.

### 2.3 TECAS Protocol

**FIGURE 1:**
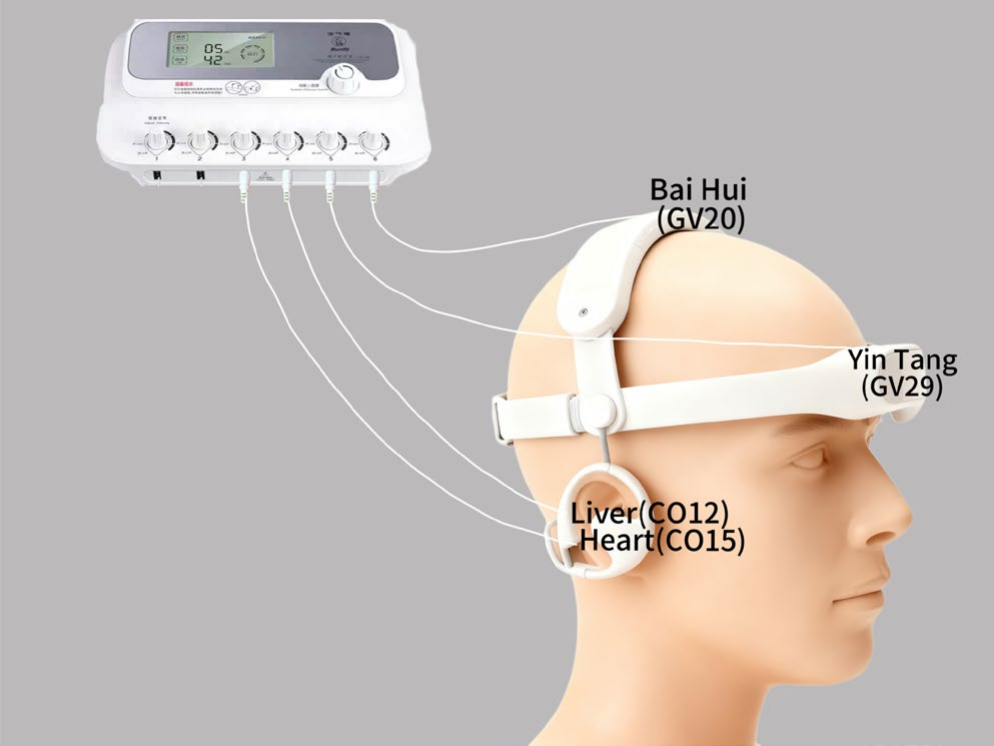
The TECAS device. The cranial acupoints: GV20 (Bai Hui) and GV29 (Yin Tang), The auricular acupoints: CO12 (liver) and CO15 (heart).

The TECAS protocol was identical to that described in our previous study [9]. Briefly, patients received stimulation twice daily (two 30‐min sessions per day, once in the morning and once in the evening; the evening treatment could be done 30 min before bedtime) for eight consecutive weeks. During each session, participants were placed in a supine or seated position and stimulation was delivered using an SDZ‐V electronic acupuncture device (Hwato, Jiangsu, China), equipped with two cranial surface electrodes and a pair of auricular clip electrodes (Figure 1).

The cranial electrodes were positioned at Baihui (GV20) and Yintang (GV29), while the auricular electrodes were attached to the Heart (CO15) and Liver (CO12) acupoints. Electrical stimulation was delivered as an asymmetric biphasic pulse with alternating frequencies of 4 Hz (5 s) and 20 Hz (10 s). Pulse width was set at 200 ms, and current intensity was individually adjusted between 0.5 and 2.0 mA according to participant tolerance.

### 2.4 EEG Acquisition

Resting‐state EEG data were acquired using a 32‐channel Electrical Geodesics system (EGI System 400), with electrodes positioned according to the international 10–20 system. Signals were recorded at a sampling rate of 1000 Hz with a resolution of 0.1 μV. Cz served as the online reference and Fz as the ground. Online band‐pass filtering was applied between 0.01 and 250 Hz, and electrode impedances were maintained below 20 kΩ.

Participants were instructed to avoid sleep deprivation, alcohol, and recreational drugs within 24 h prior to EEG recording, and to refrain from smoking, coffee, or tea consumption within 8 h before the session. A 5‐min eyes‐closed resting‐state EEG was recorded at baseline (within 1 week before TECAS initiation) and post‐treatment (within 1 week after the final TECAS session). Participants were instructed to remain relaxed yet alert, minimizing eye blinks and head movements. For subsequent analyses, data from 22 standard scalp electrodes were retained; included channels are detailed in Figure S1.

### 2.5 EEG Data Preprocessing

EEG preprocessing was conducted using MATLAB (R2013b) and EEGLAB [32], following procedures established in our prior work [31]. The preprocessing pipeline consisted of the following steps: (1) selection of 22 predefined scalp electrodes; (2) band‐pass filtering between 0.1 and 50 Hz using a zero‐phase finite impulse response (FIR) filter, with an additional notch filter at 49–51 Hz to suppress line noise; (3) identification and interpolation of bad channels using spherical interpolation; (4) removal of ocular, muscular, and cardiac artifacts via independent component analysis (ICA). Artifact components were identified using ICLabel, and components with a probability > 0.8 for eye blink, horizontal eye movement, muscle, or heartbeat were removed, followed by visual inspection; (5) segmentation of the continuous EEG into non‐overlapping 2‐s epochs; (6) rigorous visual inspection to exclude residual artifacts; (7) re‐referencing to the common average reference, including Cz; and (8) selection of 90 artifact‐free epochs per participant (180 s total) for subsequent analyses.

### 2.6 EEG Functional Connectivity Analysis

EEG FC analyses were performed using custom MATLAB scripts in conjunction with the current source density (CSD) toolbox [33, 34, 35]. A surface Laplacian transform was applied to the EEG signals to obtain CSD estimates using a spherical spline model with a smoothing constant of 10^−5^ [33, 34, 35], thereby reducing the influence of volume conduction.

FC was quantified using four complementary metrics: coherence (COH), phase‐locking value (PLV), phase lag index (PLI), and weighted phase lag index (WPLI). These analyses were calculated for the delta (1–4 Hz), theta (4–8 Hz), alpha (8–12 Hz), beta (12–30 Hz), and γ (30–45 Hz) frequency bands. COH measures the linear frequency‐specific relationship between two signals based on cross‐spectral density [36]. PLV, PLI, and WPLI quantify phase synchronization by analyzing the statistical properties of instantaneous phase differences between signals [37]. Specifically, for each epoch, signals were narrowband filtered using a zero‐phase FIR filter (order 165, transition band 1 Hz), followed by Hilbert transform to extract instantaneous phase. Phase‐based metrics were computed pointwise across each 2 s epoch (1000 time points at 500 Hz) and then averaged across the 90 epochs. Compared with COH and PLV, PLI and WPLI are less susceptible to zero‐lag artifacts and are considered more robust indicators of true functional interactions [38].

### 2.7 Statistics

Statistical analyses were performed using MATLAB (including the Network‐Based Statistic, NBS, toolbox [39]) and SPSS software. Network visualization was conducted with the BrainNet Viewer toolbox [40]. Normality of continuous variables was assessed using the Shapiro–Wilk test. Normally distributed data are presented as mean ± standard deviation, whereas non‐normally distributed data are expressed as median and interquartile range (IQR). Categorical variables were compared using chi‐square tests.

Demographic and baseline clinical variables were compared between MDD patients and HCs using independent‐samples *t*‐tests. Within the MDD group, pre‐ and post‐treatment changes in clinical scores and EEG measures were assessed using paired‐samples *t*‐tests. Treatment response was defined as a ≥ 50% reduction in HAMD‐17 score from baseline to week 8. Baseline FC and treatment‐induced changes were compared between responders and nonresponders using independent‐samples *t*‐tests.

For EEG network analyses, edge‐wise statistical comparisons were performed using independent‐ or paired‐samples *t*‐tests, as appropriate. The NBS approach was applied to control the family‐wise error rate, with a primary threshold of *p* < 0.005 at the edge level and a corrected significance threshold of *p* < 0.05 based on 10,000 permutations [20]. Pearson correlation analyses were conducted to examine associations between EEG metrics and changes in HAMD‐17 and HAMA‐14 scores. Given the exploratory nature of these analyses, multiple comparisons were controlled using the NBS framework.

### 2.8 Procedural and Ethical Considerations

The study was conducted in accordance with the Declaration of Helsinki, and written informed consent was obtained from all participants. Following rigorous review in accordance with clinical practice standards, this trial was approved by the Ethics Committee of the Institute of Acupuncture and Moxibustion, China Academy of Chinese Medical Sciences (No. 2021‐11‐26‐1‐2) and has been registered on the WHO International Clinical Trials Registry Platform—Chinese Clinical Trial Registry (ChiCTR2200063882).

## 3 Results

### 3.1 Demographic and Clinical Characteristics

**FIGURE 2:**
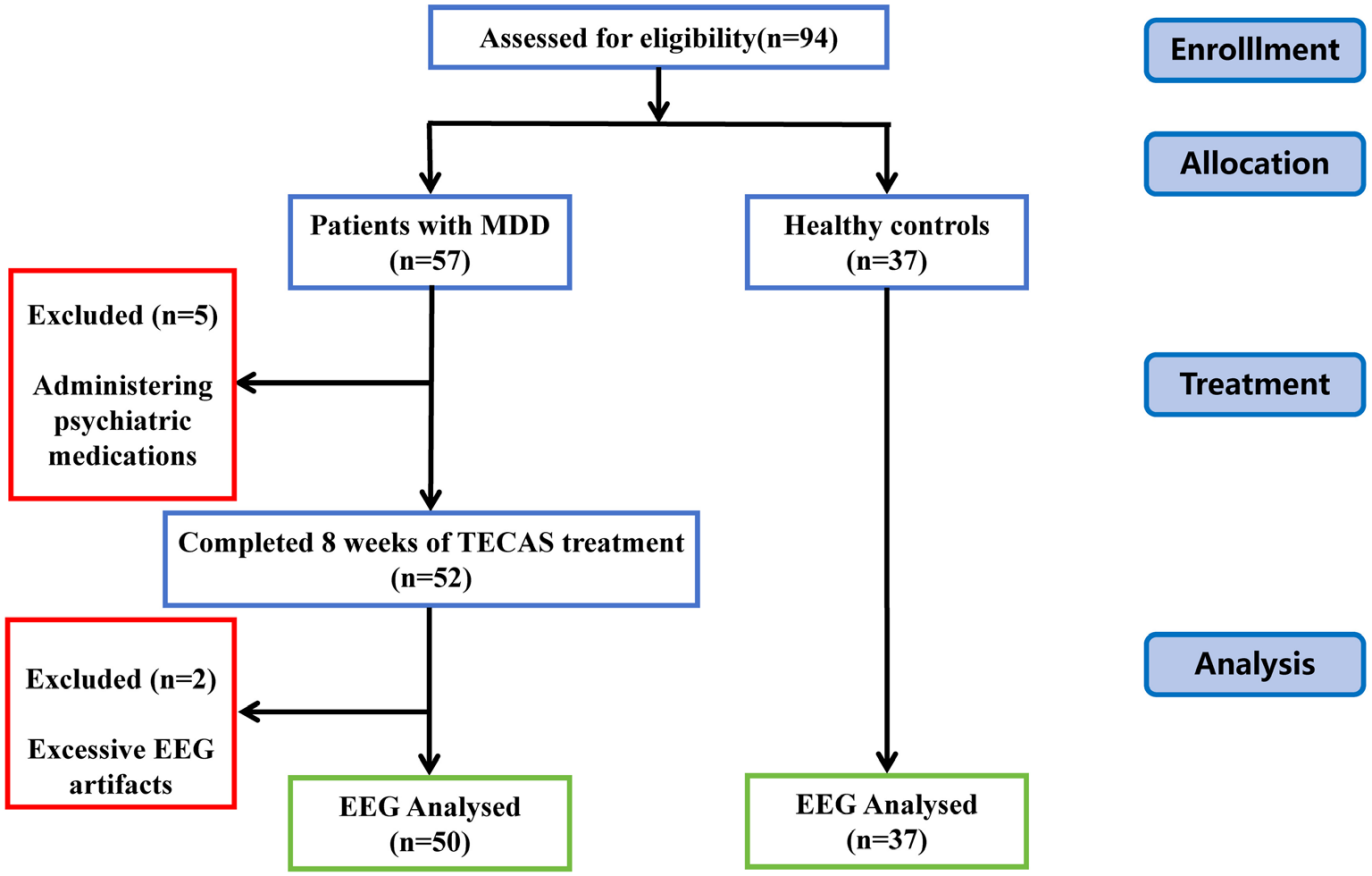
Flow diagram of individuals from enrollment to electroencephalogram (EEG) analyses. HC, healthy control; MDD major depressive disorder; TECAS, transcutaneous electrical cranial‐auricular acupoint stimulation.

A total of 57 patients with MDD and 37 HCs were initially enrolled. Five patients were excluded due to self‐administration of psychiatric medications during the study, and two were excluded because of excessive EEG artifacts (Figure 2). The final sample comprised 50 patients with MDD (12 males, 38 females; mean age 38.9 ± 13.57 years) and 37 HCs (17 males, 20 females; mean age 34.0 ± 13.26 years). No significant differences were observed between groups in age, sex distribution, or years of education (all *p* > 0.05; Table 1). As expected, baseline HAMD‐17 and HAMA‐14 scores were significantly higher in the MDD group than in HCs (both *p* < 0.001).

**TABLE 1:** Demographic information of participants.

Variable	MDD (*n* = 50)	HC (*n* = 37)	*p*
Gender (F/M)	38/12	20/17	0.082
Age (years)	38.9 ± 13.57	34.0 ± 13.26	0.100
Education (years)	15.6 ± 2.81	16.6 ± 2.49	0.085
Duration of illness (months)	10.5 ± 8.8	—	—
HAMD‐17 pre	18.3 ± 3.71	1.5 ± 1.24	< 0.001
HAMA‐14 pre	18.8 ± 5.43	2.2 ± 1.43	< 0.001

*Note:* Continuous variables are presented as mean ± standard deviation.

Abbreviations: F, female; HAMA‐14, 14‐item Hamilton Anxiety Scale; HAMD‐17, 17‐item Hamilton Depression Rating Scale; HC, healthy control; M, male; MDD, major depressive disorder.

### 3.2 Antidepressant Efficacy of TECAS

**FIGURE 3:**
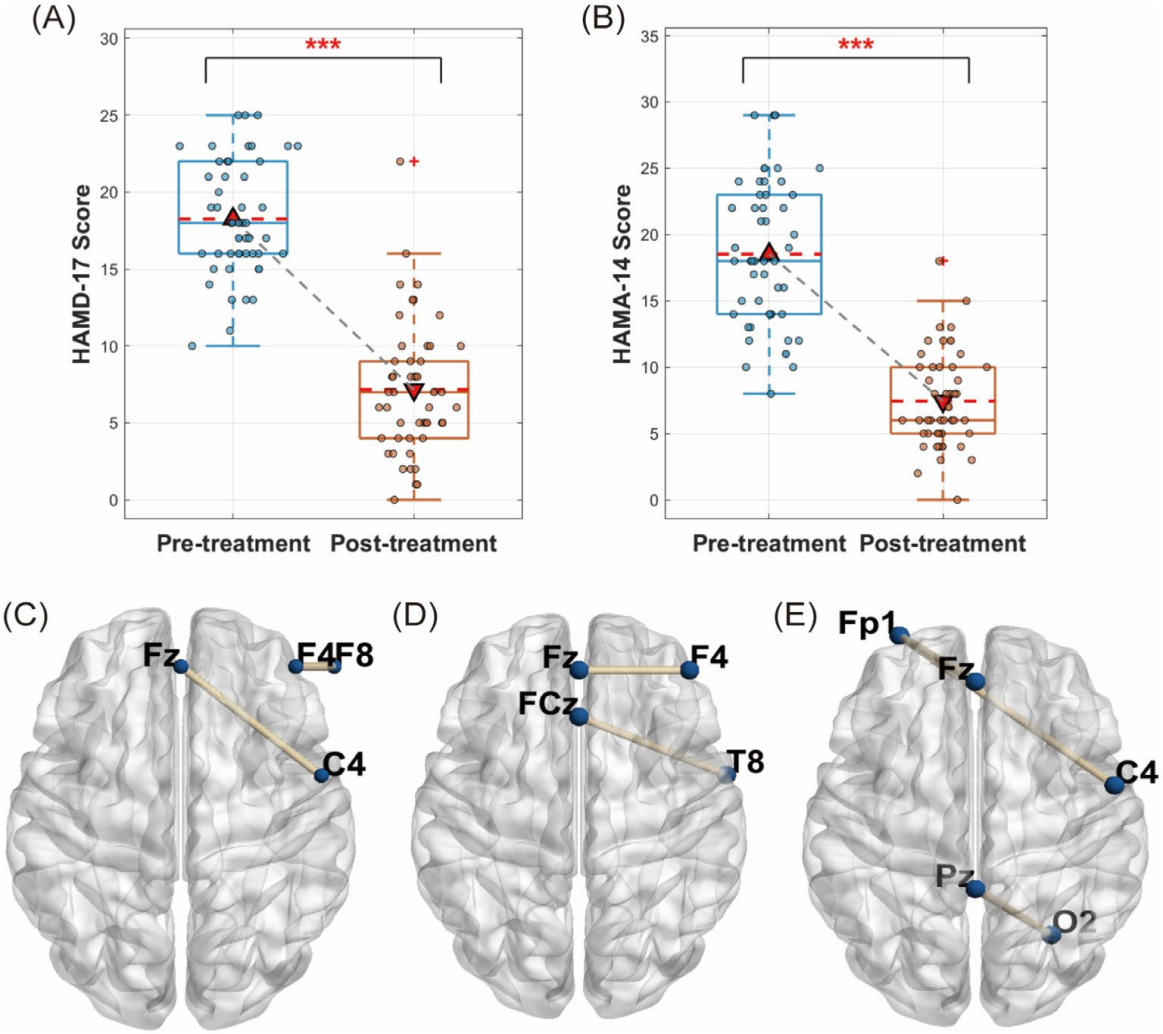
Clinical and connectivity changes (from baseline to post‐treatment) in MDD patients after TECAS treatment. (A) Changes in HAMD‐17 scale scores of MDD patients after treatment. (B) Changes in HAMA‐14 scale scores of depressed patients after treatment. (C) Compared to healthy subjects, MDD patients showed significantly increased COH strength in the δ band within the right frontoparietal region (electrode pairs Fz–C4 and F4–F8). (D) Compared to healthy subjects, MDD patients showed significantly increased COH strength in the θ band in the right frontoparietal region (electrode pairs Fz–F4 and FCz–T8). (E) After treatment, the θ‐band PLV strength in fronto‐parietal and parieto‐occipital areas (C4–Fp1, Fz–Fp1, and Pz–O2) decreased significantly in MDD patients. *Note:* ***p < 0.001, “+” indicates outliers (beyond 1.5 times the interquartile range), “▲” represents the group mean. COH, coherence; EEG, electroencephalogram; HAMA‐14, 14‐item Hamilton Anxiety Scale; HAMD‐17, 17‐item Hamilton Depression Rating Scale; MDD, major depressive disorder; TECAS, transcutaneous electrical cranial‐auricular acupoint stimulation.

After 8 weeks of TECAS treatment, patients with MDD exhibited significant reductions in both HAMD‐17 and HAMA‐14 scores (Figure 3A,B). Based on the predefined response criterion, 30 of the 50 patients (60%) were classified as responders (Figure 2). No significant difference was observed between responders and nonresponders with respect to demographic variables or baseline depression and anxiety severity (all *p* > 0.05; Table 2).

**TABLE 2:** Baseline characteristics of responders and nonresponders.

Variable	Responder (*n* = 30)	Nonresponder (*n* = 20)	*p*
Gender (F/M)	21/9	15/5	0.700
Age (years)	36.13 ± 14.06	43.00 ± 12.00	0.051
Education (years)	16.00 ± 2.57	15.00 ± 3.11	0.156
Duration of illness (months)	9.37 ± 9.92	12.20 ± 7.41	0.085
HAMD‐17 pre	19.13 ± 3.53	16.9 ± 3.66	0.064
HAMA‐14 pre	18.57 ± 5.51	18.40 ± 5.45	0.874

*Note:* Continuous variables are presented as mean ± standard deviation.

Abbreviations: F, female; HAMA‐14, 14‐item Hamilton Anxiety Scale; HAMD‐17, 17‐item Hamilton Depression Rating Scale; M, male.

### 3.3 EEG FC Features Associated With MDD Diagnosis

#### 3.3.1 Group Differences Between MDD Patients and HCs

Using the COH metric, patients with MDD demonstrated significantly increased FC within the right frontoparietal network compared with HCs. This hyperconnectivity was observed in the δ band (at an edge‐level threshold of *t* > 2.645, NBS identified two significant edges: Fz–C4, F4–F8; *p* = 0.024; see Figure 3C for details) and θ band (at an edge‐level threshold of *t* > 2.645, Fz–F4, FCz–T8; *p* = 0.024; Figure 3D), and exhibited marked right‐hemispheric asymmetry.

#### 3.3.2 Associations Between Baseline FC and Symptom Severity

No significant correlations were identified between the COH features differentiating MDD patients from HCs and baseline HAMD‐17 or HAMA‐14 scores (*p* > 0.05). Exploratory analyses across all electrode pairs revealed that baseline α‐ and β‐band FC, measured by PLI and PLV, was positively correlated with anxiety severity, particularly between frontal and occipital regions (e.g., F7–P8; Figures S2–S5). No significant correlation was observed between baseline FC and depression severity (*p* > 0.05).

### 3.4 Changes in EEG FC Following TECAS

#### 3.4.1 Treatment‐Induced Modulation of FC

Following TECAS treatment, patients exhibited a significant reduction in θ‐band PLV, primarily involving frontoparietal and parieto‐occipital connections (at an edge‐level threshold of *t* > 2.680, C4–Fp1, Fz–Fp1, Pz–O2; *p* = 0.044; Figure 3E).

#### 3.4.2 Correlations Between Connectivity Changes and Clinical Improvement

Changes in α‐band PLI and WPLI between right parietotemporal regions and frontal, temporal, and occipital areas were significantly positively correlated with reductions in HAMD‐17 scores (PLI, average *r* = 0.446, *p* = 0.017; WPLI, average *r* = 0.413, *p* = 0.012; Figures S6 and S7). No significant correlation was observed between FC changes and anxiety reduction (all *p* > 0.05).

### 3.5 EEG FC Associated With TECAS Response

#### 3.5.1 Differential Connectivity Changes in Responders and Nonresponders

**FIGURE 4:**
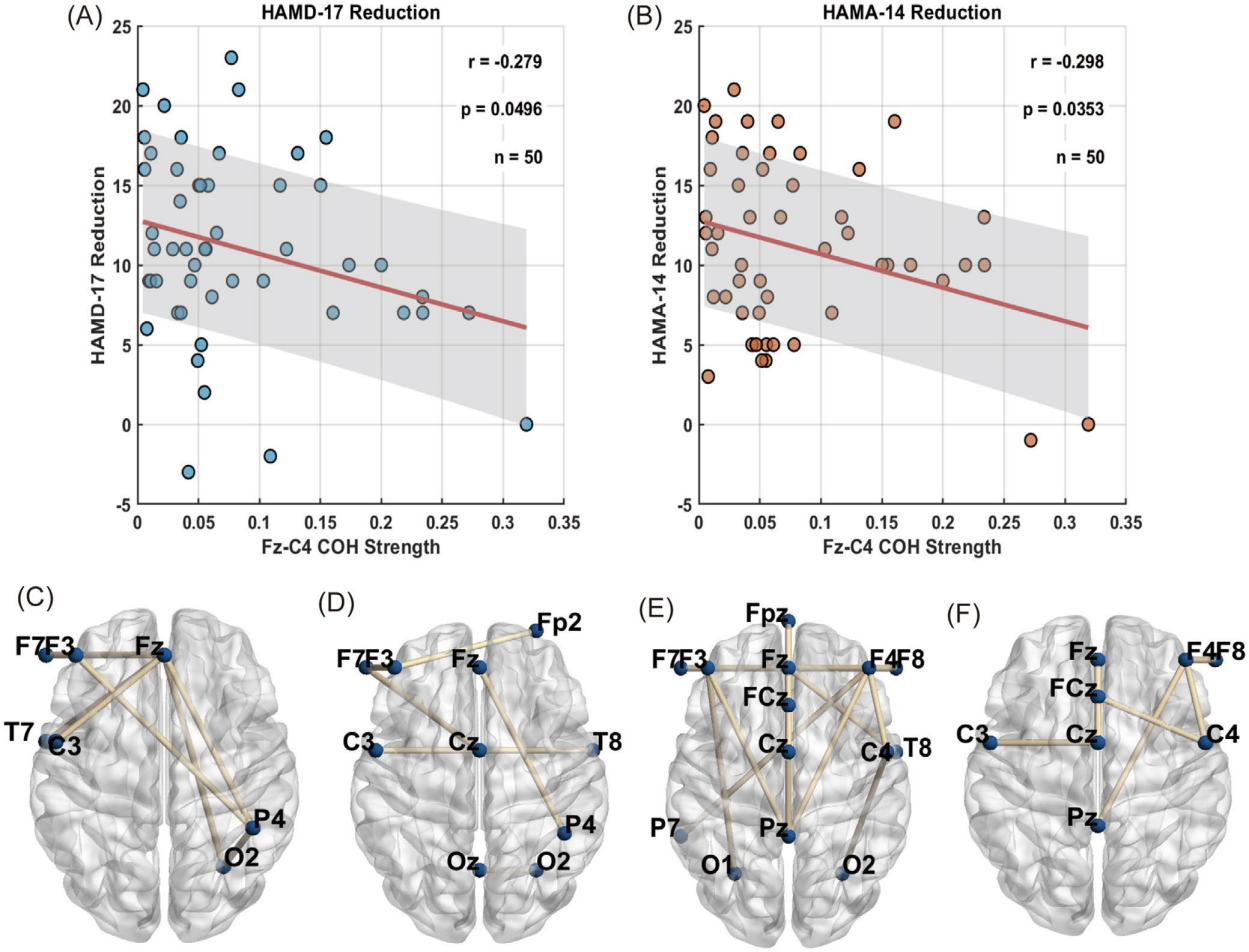
Associations between baseline EEG functional connectivity and clinical improvement, and its predictive value for TECAS treatment response. (A, B) Baseline COH strength in the δ band at the Fz–C4 electrode pair in MDD patients was significantly negatively correlated with the reduction in both HAMD‐17 scores and HAMA‐14 scores. (C) Nonresponders exhibited a significantly greater reduction in α‐band PLV compared to responders. (D) Nonresponders demonstrated a significantly greater reduction in δ‐band PLV compared to responders. (E, F) Baseline PLI and WPLI strength in the β band was significantly higher in responders compared to nonresponders. *Note:* The values in the heatmap represent the correlation coefficient (*r*). The asterisks (*) below the heatmap indicate leads that are statistically significant after multiple comparison correction. COH, coherence; EEG, electroencephalogram; HAMA‐14, 14‐item Hamilton Anxiety Scale; HAMD‐17, 17‐item Hamilton Depression Rating Scale; MDD, major depressive disorder; PLI, phase lag index; PLV, phase locking value; TECAS, transcutaneous electrical cranial‐auricular acupoint stimulation; WPLI, weighted phase lag index.

Compared with responders, nonresponders exhibited greater reductions in α‐band PLV between frontal/frontotemporal regions and distributed cortical areas (at an edge‐level threshold of *t* > 2.682, *p* = 0.038; Figure 4C), as well as greater reductions in δ‐band PLV within frontoparietal and parietal networks (at an edge‐level threshold of *t* > 2.682, *p* = 0.049; Figure 4D).

#### 3.5.2 Baseline FC Correlated With Clinical Improvement

Baseline δ‐band COH between Fz and C4 was negatively correlated with reductions in both HAMD‐17 (*r* = −0.279, *p* = 0.0496) and HAMA‐14 scores (*r* = −0.298, *p* = 0.035; Figure 4A,B), indicating that lower baseline δ‐band COH was associated with greater improvement. Exploratory analyses further revealed that higher baseline α‐band and γ‐band connectivity across parietal, frontal, temporal, and occipital regions predicted greater improvements in anxiety symptoms (Figures S8–S12). Moreover, higher baseline α‐band COH strength across the left frontal and parietal regions predicted greater improvements in depressive symptoms (Figure S13). To further explore the potential stratification utility of baseline FC, we conducted an analysis with depression reduction as the dependent variable and anxiety reduction as a covariate. The direct effect of most baseline FC measures on depression reduction was no longer significant (except for the Cz–F3 electrode pair in the α‐band COH, $F_{\left(1,47\right)}$ = 5.519, *p* = 0.023, *η* ^2^ = 0.105), whereas anxiety reduction significantly moderated the effect of baseline FC on depression reduction (all *p* < 0.05, see Tables S1 and S2).

Responders also exhibited significantly stronger baseline β‐band phase synchronization, as measured by PLI (at an edge‐level threshold of *t* > 2.682, *p* = 0.011, Figure 4E) and WPLI (at an edge‐level threshold of *t* > 2.682, *p* = 0.028, Figure 4F), compared with nonresponders. After controlling for baseline anxiety as a covariate, responders still exhibited significantly higher baseline PLI and WPLI strength than nonresponders. Except for the O2‐C4 electrode pair (in the PLI metric, $F_{\left(1,47\right)}$ = 4.627, *p* = 0.037, *η* ^2^ = 0.090), baseline anxiety level did not significantly moderate the effect of baseline PLI and WPLI strength on treatment response (all *p* > 0.05, see Tables S3 and S4).

## 4 Discussion

This study provides the first systematic evidence that EEG‐based FC features may serve as potential biomarkers for predicting therapeutic response to TECAS in MDD. Our findings indicate that aberrant connectivity within the right frontoparietal network, particularly in slow‐wave (δ/θ) and β frequency bands, characterizes the pathophysiology of MDD and is closely linked to clinical response to TECAS. Consequently, these network features may represent promising neurophysiological biomarkers for guiding personalized TECAS therapy.

### 4.1 Neuroregulatory Mechanisms Underlying TECAS

The antidepressant effects of noninvasive neuromodulation techniques, including auricular vagus nerve stimulation and trigeminal nerve stimulation, have been increasingly supported by clinical evidence [8, 9, 10, 11]. TECAS integrates cranial and auricular stimulation, engaging afferent pathways of both the trigeminal and vagus nerves. These signals converge at the spinal trigeminal nucleus and nucleus tractus solitarius, subsequently modulating neuromodulatory nuclei such as the locus coeruleus and dorsal raphe nucleus [41, 42]. Analogous to transcutaneous vagus and trigeminal nerve stimulation [41], this shared ascending pathway provides a neuroanatomical foundation for the widespread network‐level effects observed following TECAS [12, 13].

### 4.2 Modulation of Aberrant Brain Networks by TECAS

Consistent with prior studies, patients with MDD exhibited enhanced slow‐wave synchronization within the right frontoparietal network, a pattern associated with impaired cognitive control and maladaptive affective processing [10, 11, 24, 43]. TECAS treatment significantly reduced θ‐band synchronization within frontocentral networks, and we propose the hypothesis that TECAS may normalize pathological slow‐wave activity [44, 45, 46]. Importantly, greater clinical improvement was associated with enhanced α‐band synchronization following treatment, consistent with restored large‐scale network coordination and adaptive neuroplasticity [10, 12, 13, 14].

### 4.3 Frequency‐Specific Brain Networks Associated With TECAS Efficacy

#### 4.3.1 Prognostic Significance of Slow‐Wave Bands (δ/θ)

We observed that MDD patients exhibited enhanced δ/θ band connectivity compared to HCs, and this abnormality was modulated after TECAS treatment. Crucially, lower pretreatment δ‐band connectivity strength in the right frontoparietal network predicted greater clinical improvement; however, analysis of covariance revealed that this effect was influenced by anxiety factors. Previous studies have reported inconsistent findings regarding the predictive role of slow‐wave band FC for antidepressant response, which may be attributable to the complexity of MDD populations [47, 48]. While some studies link enhanced slow‐wave activity to poor drug efficacy [26], others associate it with a positive response [25, 27]. Given that anxiety influenced the predictive effect of pretreatment δ‐band connectivity, we hypothesize that TECAS normalizes slow‐wave activity in part via its anxiolytic action.

#### 4.3.2 The Dual Role of the α Band

We found that the enhancement of α‐band connectivity following TECAS treatment correlated with overall symptom improvement. Moreover, higher baseline α‐band connectivity specifically predicted a better anti‐anxiety response. This indicates that pre‐existing α synchrony, while indicative of a pathological state, may also reflect a preserved neuroplasticity reserve, rendering the brain more amenable to adaptive reorganization under TECAS intervention [20, 44, 49]. Therefore, α‐band connectivity might be better regarded as a state‐dependent characteristic rather than a mere pathological marker.

#### 4.3.3 Unique Predictive Value of the β Band

While showing a positive correlation with baseline anxiety, stronger pretreatment β‐band phase synchrony characterized TECAS responders. Given that β‐band activity has been linked to cortical hyperarousal and anxious reactivity, we propose that TECAS may target anxiety‐related high‐frequency brain electrical activity. This mechanism is consistent with observations in other neuromodulation studies where high baseline β‐band brain activity sometimes predicts better efficacy [28]. The lack of a significant correlation with HAMD‐17 scores could be due to the high comorbidity of anxiety in our depressed cohort, which makes it difficult to disentangle the two conditions. In addition, it is also possible that the initial therapeutic effects of TECAS are mediated by its anxiolytic properties [50], which secondarily contribute to the alleviation of depressive symptoms, and this hypothesis is supported by relevant studies on transcranial electrical stimulation [51]. Nevertheless, our analysis with anxiety as a covariate showed that this indicator remained relatively robust for predicting antidepressant response.

### 4.4 Clinical Significance and Limitations

This study represents the first clinical exploration utilizing EEG‐based FC to predict therapeutic response to TECAS in MDD. By addressing the challenge of significant interindividual variability in treatment outcomes, our work provides preliminary electrophysiological evidence for stratifying patients and identifying those most likely to benefit from TECAS. Several limitations of this study should be acknowledged. First, the limited electrode density (only 22 electrodes applied) may have restricted the accuracy of the study, warranting further verification with high‐density EEG and functional magnetic resonance imaging (fMRI). Second, the prospective single‐arm trial aimed to investigate FC differences between responders and nonresponders and was not designed to include a control group. Third, the modest sample size and the frequent comorbidity of depressive and anxiety symptoms may have compromised the accuracy of the results. Future research should seek to optimize and validate this biomarker framework by: (1) expanding the cohort size; (2) implementing more refined, dimensional symptom assessments; (3) setting up a control group; (4) incorporating high‐density EEG/fMRI, and (5) conducting dynamic follow‐up.

## 5 Conclusions

This study suggests that EEG‐based FC may serve as potential biomarkers associated with antidepressant response to TECAS. Specifically, enhanced pretreatment β‐band connectivity and post‐treatment reduction in frontoparietal δ‐band connectivity were correlated with symptom improvement. These results provide preliminary neuroelectrophysiological evidence for forecasting TECAS efficacy, which could inform the development of personalized neuromodulation strategies for depression. Future research with larger cohorts and refined analytics is needed to translate these biomarkers into clinically actionable tools for precision psychiatry.

## Author Contributions

**Yanan Zhao:** methodology, investigation, formal analysis, data curation, writing – review and editing. **Zhouzhi Yin:** methodology, investigation, formal analysis, writing – original draft. **Ying Zhang:** data curation, visualization, writing – original draft. **Xiaoqiu Wang** and **Chengyong Liu:** project administration, supervision. **Wenzhong Wu** and **Peijing Rong:** conceptualization, methodology, resources, supervision, and funding acquisition.

## Funding

This work was supported by the National Natural Science Foundation of China (No. 82405584), Major Brain Disorders Research Program of the Capital's Health Development Research Fund (No. 2024‐1‐4011), Scientific and Technological Innovation Project of China Academy of Chinese Medical Sciences (No. ZZ17‐YQ‐031; No. CI2023C003YG; CIZJS2025022; ZZCZ2025‐032) and Postgraduate Research & Practice Innovation Program of Jiangsu Province (No. SJCX25_0940).

## Conflicts of Interest

The authors declare no conflicts of interest.

## Supporting information


**Figure S1:** EEG electrodes included and their topography.
**Figure S2:** Correlation analysis between baseline functional connectivity PLV and clinical scales in MDD patients.
**Figure S3:** Correlation analysis between baseline functional connectivity PLV and clinical scales in MDD patients.
**Figure S4:** Correlation analysis between baseline functional connectivity PLI and clinical scales in MDD patients.
**Figure S5:** Correlation analysis between baseline functional connectivity PLI and clinical scales in MDD patients.
**Figure S6:** Correlation analysis between changes in EEG functional connectivity PLI and clinical scales in MDD patients after TECAS treatment.
**Figure S7:** Correlation analysis between changes in EEG functional connectivity WPLI and clinical scales in MDD patients after TECAS treatment.
**Figure S8:** Correlation analysis between baseline EEG functional connectivity COH in MDD patients and changes in clinical scales following TECAS treatment.
**Figure S9:** Correlation analysis between baseline EEG functional connectivity PLI in MDD patients and changes in clinical scales following TECAS treatment.
**Figure S10:** Correlation analysis between baseline EEG functional connectivity PLV in MDD patients and changes in clinical scales following TECAS treatment.
**Figure S11:** Correlation analysis between baseline EEG functional connectivity WPLI in MDD patients and changes in clinical scales following TECAS treatment.
**Figure S12:** Correlation analysis between baseline EEG functional connectivity WPLI in MDD patients and changes in clinical scales following TECAS treatment.
**Figure S13**: Correlation analysis between baseline EEG functional connectivity COH in MDD patients and changes in clinical scales following TECAS treatment.
**Table S1:** Anxiety‐adjusted baseline functional connectivity (α‐band COH metric) and depression reduction.
**Table S2:** Anxiety‐adjusted baseline functional connectivity (δ‐band COH metric) and depression reduction.
**Table S3:** Anxiety‐adjusted baseline functional connectivity (β‐band PLI metric) associated with treatment response.
**Table S4:** Anxiety‐adjusted baseline functional connectivity (β‐band WPLI metric) associated with treatment response.

## Data Availability

The data that support the findings of this study are available from the corresponding author upon reasonable request.
