# Supplementary material for: EEG Functional Connectivity Associated With Antidepressant Response to Transcutaneous Electrical Cranial‐Auricular Acupoint Stimulation
Source: CNS Neurosci Ther. 2026 Jul 1;32(7):e70920. doi: 10.1002/cns.70920 (PMC13323169; doi:10.1002/cns.70920)
Supplement: Supplementary file 1 — Figure S1: EEG electrodes included and their topography. Figure S2: Correlation analysis between baseline functional connectivity PLV and clinical scales in MDD patients. Figure S3: Correlation analysis between baseline functional connectivity PLV and clinical scales in MDD patients. Figure S4: Correlation analysis between baseline functional connectivity PLI and clinical scales in MDD patients. Figure S5: Correlation analysis between baseline functional connectivity PLI and clinical scales in MDD patients. Figure S6: Correlation analysis between changes in EEG functional connectivity PLI and clinical scales in MDD patients after TECAS treatment. Figure S7: Correlation analysis between changes in EEG functional connectivity WPLI and clinical scales in MDD patients after TECAS treatment. Figure S8: Correlation analysis between baseline EEG functional connectivity COH in MDD patients and changes in clinical scales following TECAS treatment. Figure S9: Correlation analysis between baseline EEG functional connectivity PLI in MDD patients and changes in clinical scales following TECAS treatment. Figure S10: Correlation analysis between baseline EEG functional connectivity PLV in MDD patients and changes in clinical scales following TECAS treatment. Figure S11: Correlation analysis between baseline EEG functional connectivity WPLI in MDD patients and changes in clinical scales following TECAS treatment. Figure S12: Correlation analysis between baseline EEG functional connectivity WPLI in MDD patients and changes in clinical scales following TECAS treatment. Figure S13: Correlation analysis between baseline EEG functional connectivity COH in MDD patients and changes in clinical scales following TECAS treatment. Table S1: Anxiety‐adjusted baseline functional connectivity (α‐band COH metric) and depression reduction. Table S2: Anxiety‐adjusted baseline functional connectivity (δ‐band COH metric) and depression reduction. Table S3: Anxiety‐adjusted baseline functional connect [file CNS-32-e70920-s001.docx]

**Content**

Supplementary Figure 1. EEG electrodes included and their topography.........................................2

[Supplementary Figure 2-5. Correlation Analysis between Baseline Functional Connectivity and Clinical Scales in Depressive Patients. 3](#_Toc1433237693)

Supplementary Figure 6-7. Correlation Analysis Between Changes in EEG Functional Connectivity and Clinical Scales in MDD Patients After TECAS Treatment...................................7

Supplementary Figure 8-13. Correlation Analysis between Baseline EEG Functional Connectivity in MDD Patients and Changes in Clinical Scales Following TECAS Treatment..............................9

Supplementary Table 1. Anxiety‑Adjusted Baseline Functional Connectivity (α-band COH metric) and Depression Reduction...............................................................................................................15

Supplementary Table 2. Anxiety‑Adjusted Baseline Functional Connectivity (δ-band COH metric) and Depression Reduction...............................................................................................................16

Supplementary Table 3. Anxiety‑Adjusted Baseline Functional Connectivity (β-band PLI metric) Associated with Treatment Response .............................................................................................17

Supplementary Table 4. Anxiety‑Adjusted Baseline Functional Connectivity (β-band WPLI metric) Associated with Treatment Response .............................................................................................18

**Supplementary Figure 1. EEG electrodes included and their topography**

22 channels (Fp1, Fp2, F3, F4, C3, C4, P3, P4, O1, O2, F7, F8, T7, T8, P7, P8, Fz, Pz, Oz, Fpz, FCz, Cz) of EEG data were retained for each subject for subsequent analysis.


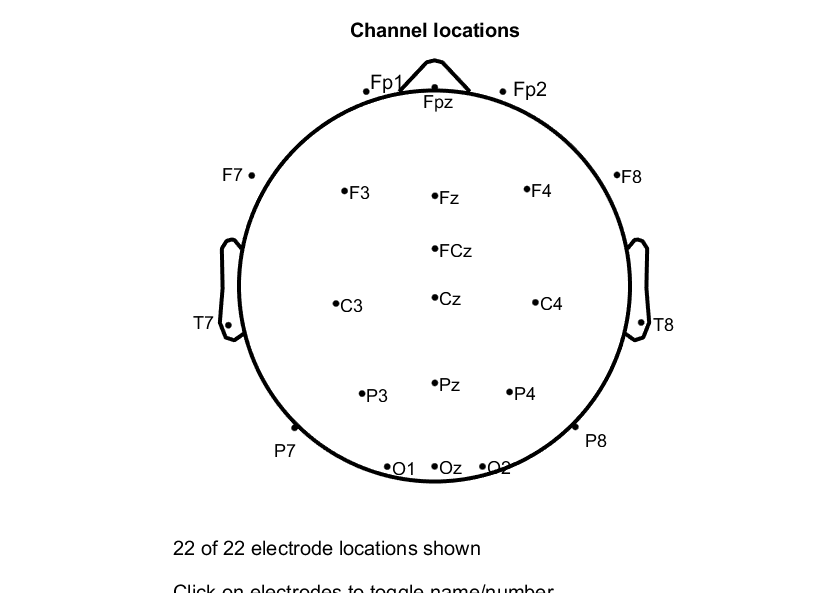


**Supplementary Figure 2. Correlation Analysis between Baseline Functional Connectivity PLV and Clinical Scales in MDD Patients**

The functional connectivity strength of PLV in the α band between the frontal and occipital lobes (the Cz-P3, FCz-P7, P8-F8, P8-T8, P7-F3, P7-F7 electrode pair) in MDD patients showed positive correlations with the HAMA-14 scale scores (average r = 0.418, *P* = 0.033). The values in the heatmap represent the correlation coefficient (r). The asterisks (*) below the heatmap indicate leads that are statistically significant after multiple comparison correction.


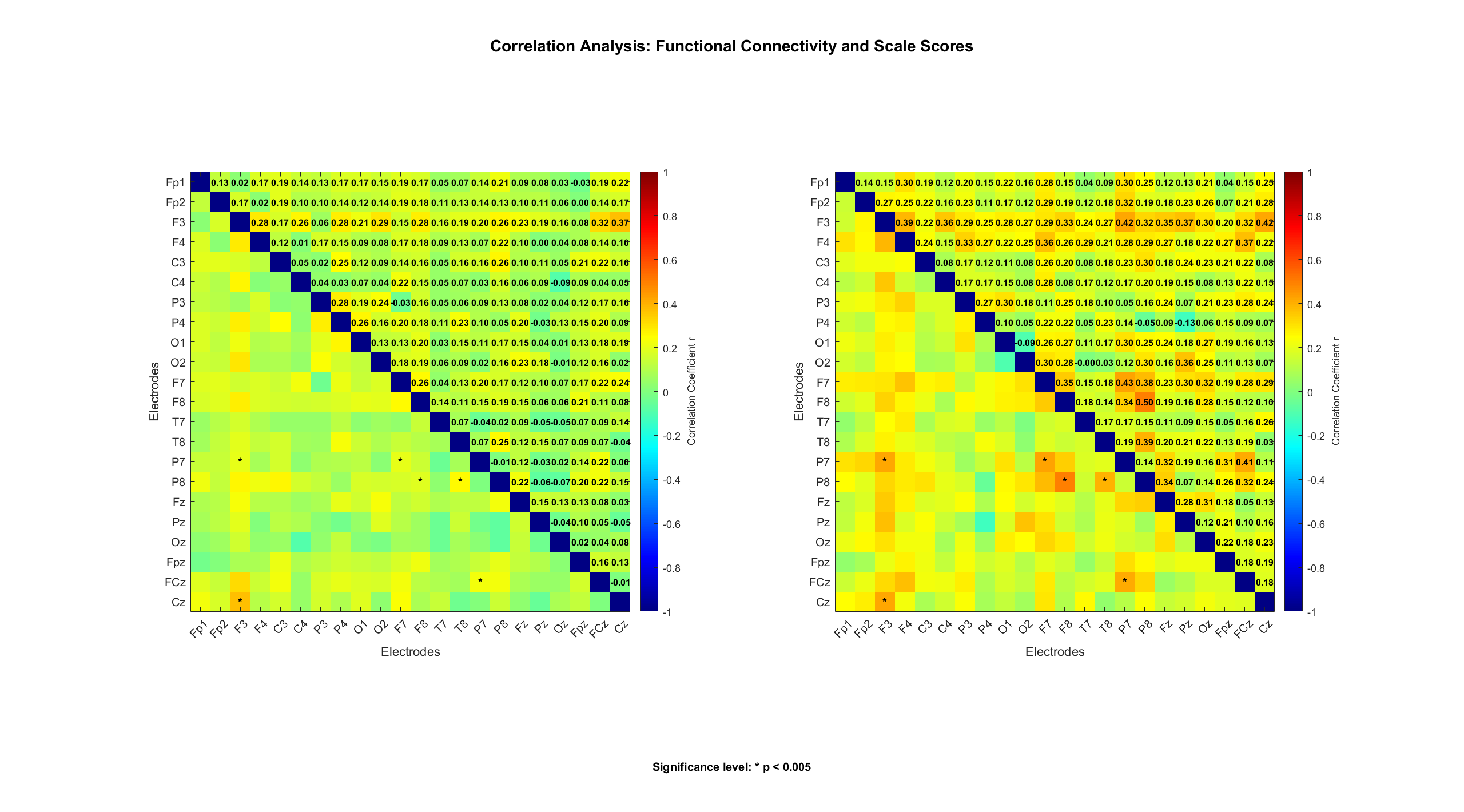


**Supplementary Figure 3. Correlation Analysis between Baseline Functional Connectivity PLV and Clinical Scales in MDD Patients**

The functional connectivity strength of PLV in the β band between the frontal and occipital lobes (the Cz-F3, FCz-P7, FPz-P7, F8-P8, P7-F4, F7-O2 electrode pair) in MDD patients showed positive correlations with the HAMA-14 scale scores (average r = 0.454, *P* = 0.031). The values in the heatmap represent the correlation coefficient (r). The asterisks (*) below the heatmap indicate leads that are statistically significant after multiple comparison correction.


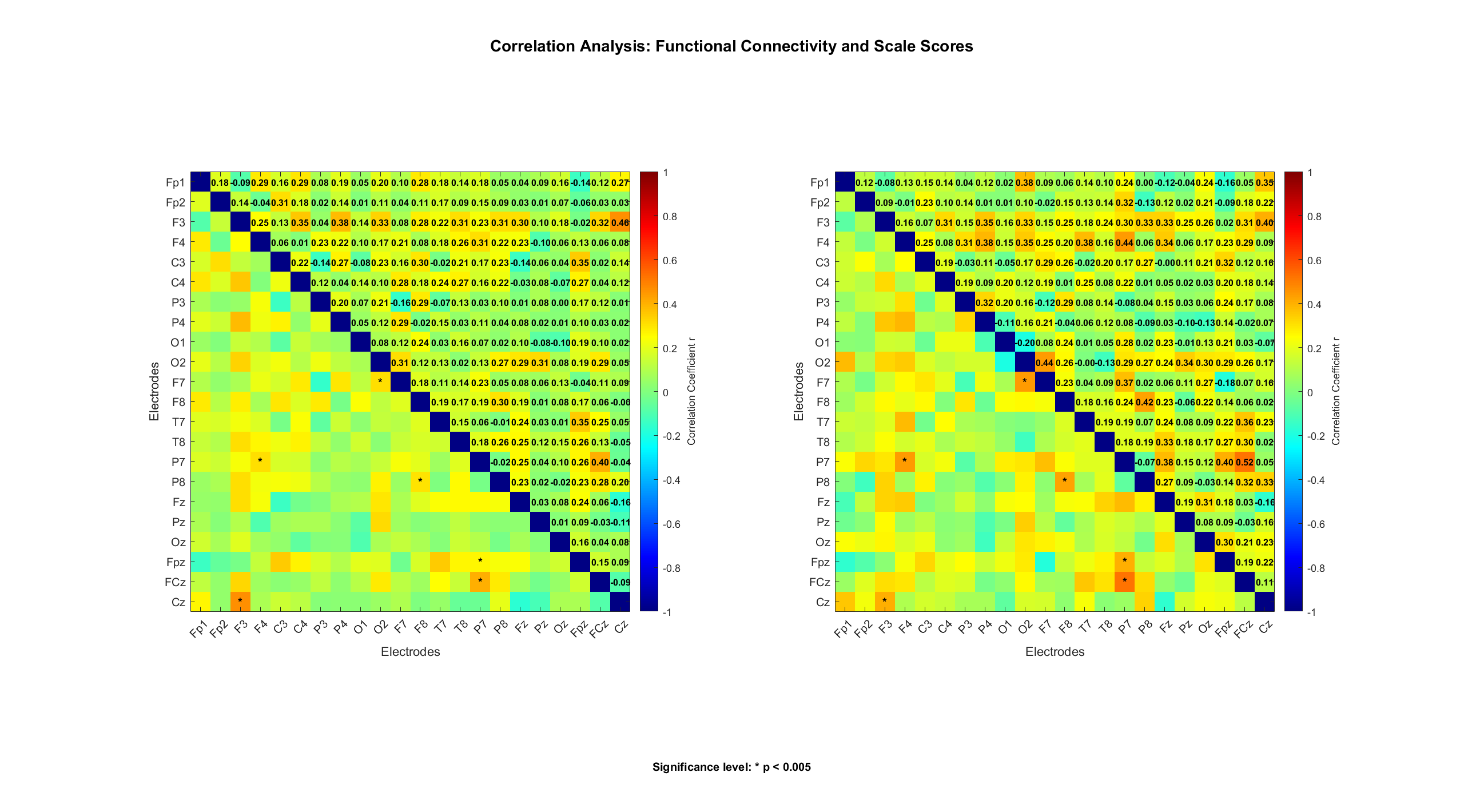


**Supplementary Figure 4. Correlation Analysis between Baseline Functional Connectivity PLI and Clinical Scales in MDD Patients**

The functional connectivity strength of PLI in the α band between the frontal and occipital lobes (the Oz-P4, Oz-P3, Oz-F8, F8-O1, F8-O2, F7-O2, F7-P8 electrode pair) in MDD patients showed positive correlations with the HAMA-14 scale scores (average r = 0.416, *P* = 0.017). The values in the heatmap represent the correlation coefficient (r). The asterisks (*) below the heatmap indicate leads that are statistically significant after multiple comparison correction.


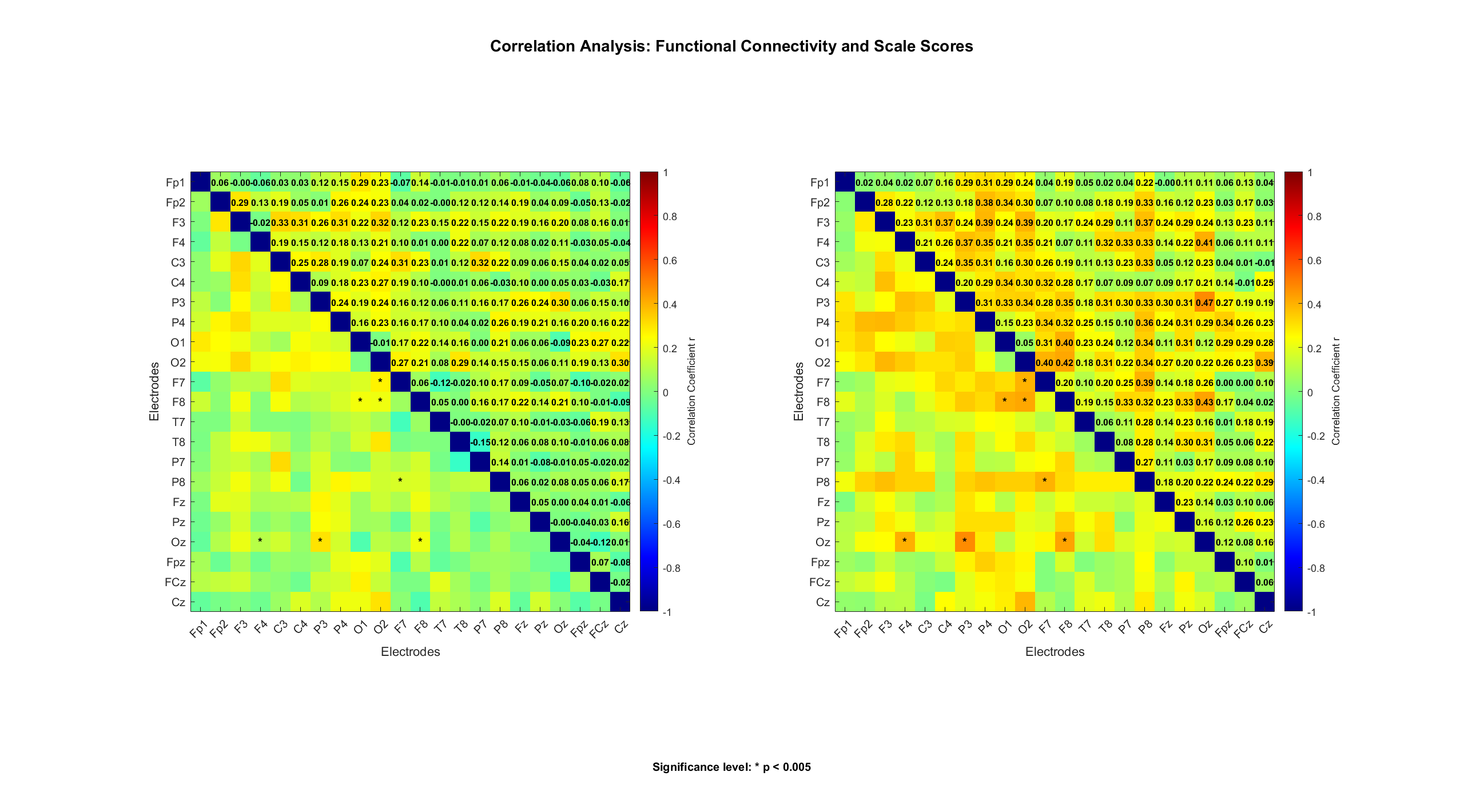


**Supplementary Figure 5. Correlation Analysis between Baseline Functional Connectivity PLI and Clinical Scales in MDD Patients**

The functional connectivity strength of PLI in the β band between the frontal and occipital lobes (the Oz-P4, Oz-P3, Oz-F8, F8-O1, F8-O2, F7-O2, F7-P8 electrode pair) in MDD patients showed positive correlations with the HAMA-14 scale scores (average r = 0.408, *P* = 0.032). The values in the heatmap represent the correlation coefficient (r). The asterisks (*) below the heatmap indicate leads that are statistically significant after multiple comparison correction.


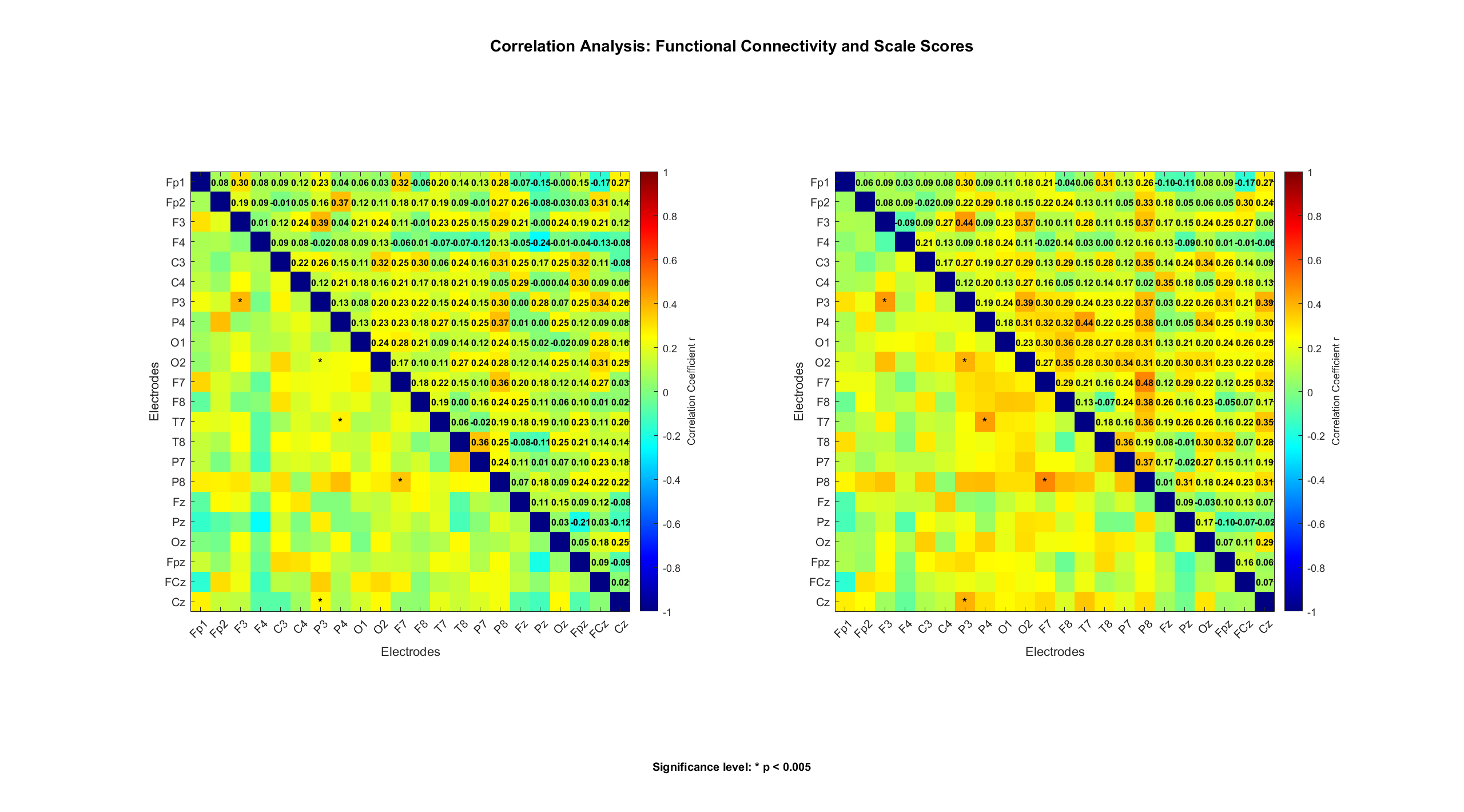


**Supplementary Figure 6. Correlation Analysis Between Changes in EEG Functional Connectivity PLI and Clinical Scales in MDD Patients After TECAS Treatment**

Changes in α-band PLI between the right parietotemporal areas (the C3-F3, C3-P4, C3-P8, P4-O2, P4-P8, Fz-P4, O1-FCz electrode pair) and the frontal, temporal, and occipital lobes were significantly positively correlated with changes in HAMD-17 scores (average r = 0.446, *P* = 0.017). The values in the heatmap represent the correlation coefficient (r). The asterisks (*) below the heatmap indicate leads that are statistically significant after multiple comparison correction.


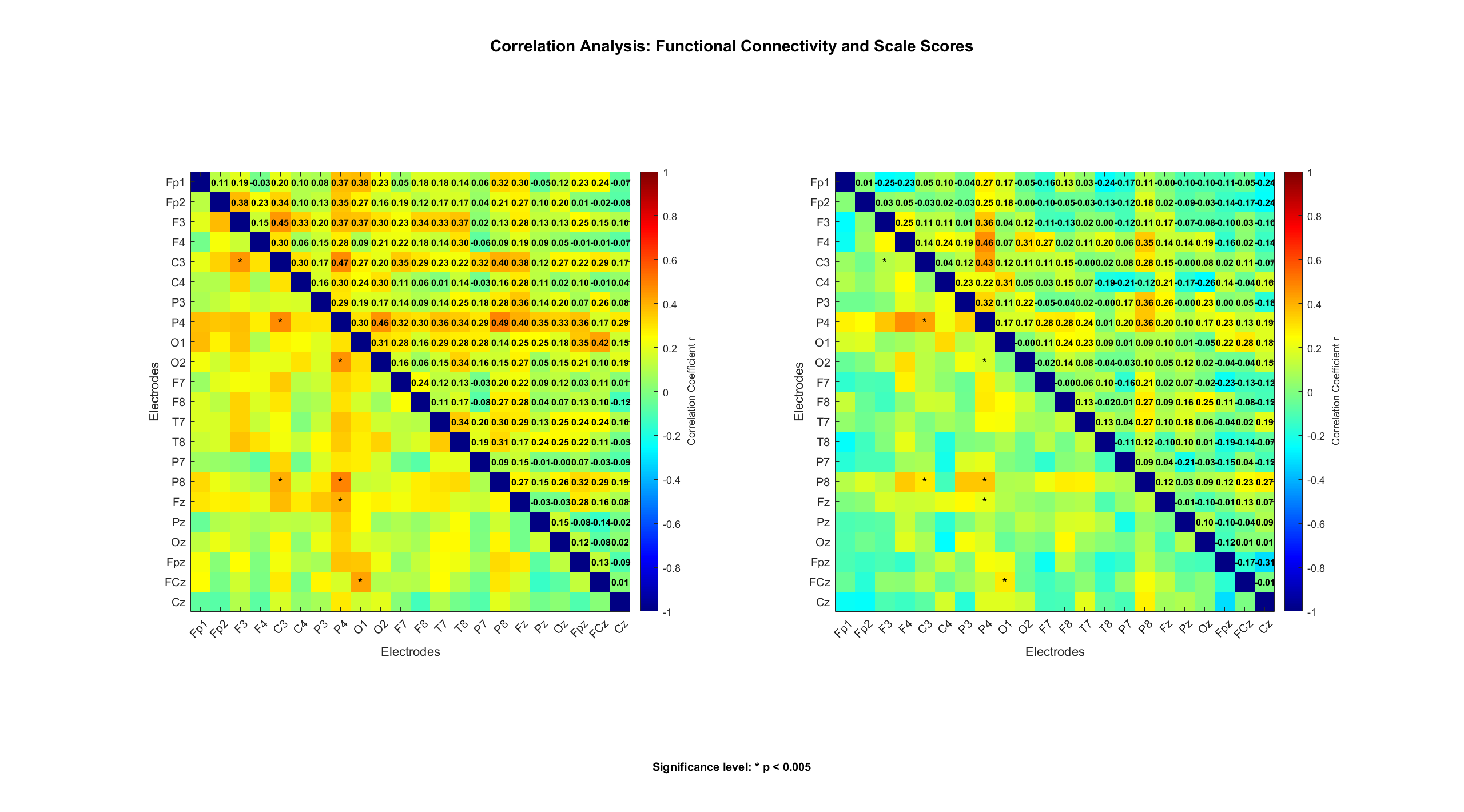


**Supplementary Figure 7. Correlation Analysis Between Changes in EEG Functional Connectivity WPLI and Clinical Scales in MDD Patients After TECAS Treatment**

Changes in α-band WPLI between the right parietotemporal areas (the C3-F3, O1-F3, P8-C3, P4-C3, P4-P8, Fz-P4, O2-P4 electrode pair) and the frontal, temporal, and occipital lobes were significantly positively correlated with changes in HAMD-17 scores (average r = 0.413, *P* = 0.012). The values in the heatmap represent the correlation coefficient (r). The asterisks (*) below the heatmap indicate leads that are statistically significant after multiple comparison correction.


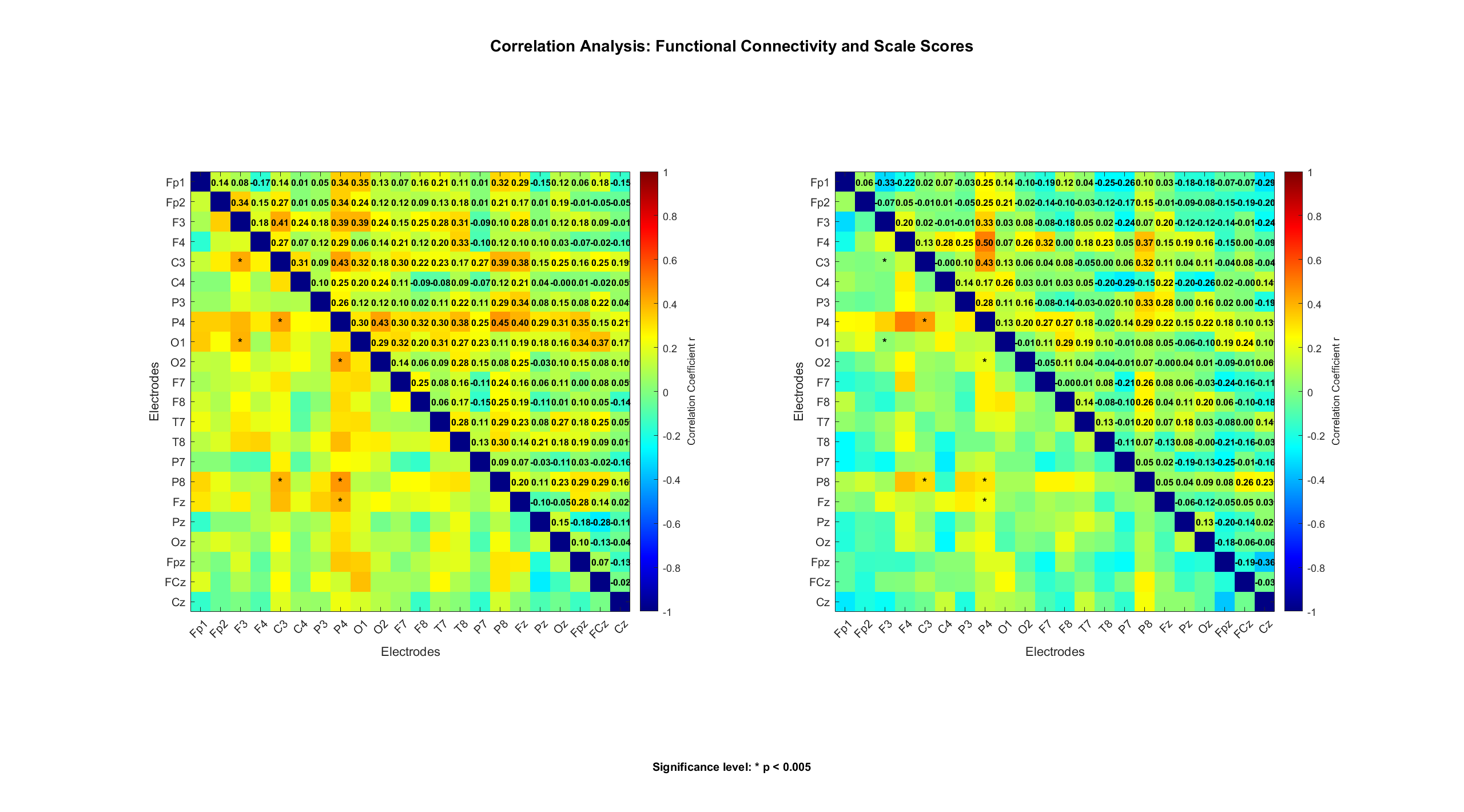


**Supplementary Figure 8. Correlation Analysis between Baseline EEG Functional Connectivity COH in MDD Patients and Changes in Clinical Scales Following TECAS Treatment**

The baseline α-band COH strength between parietal areas and frontal, occipital, and temporal areas (Cz-F3, P7-F3, F7-F4, P7-O1, P7-F7) was significantly positively correlated with the change in HAMA-14 scores (average r = 0.424, *P* = 0.021). The values in the heatmap represent the correlation coefficient (r). The asterisks (*) below the heatmap indicate leads that are statistically significant after multiple comparison correction.


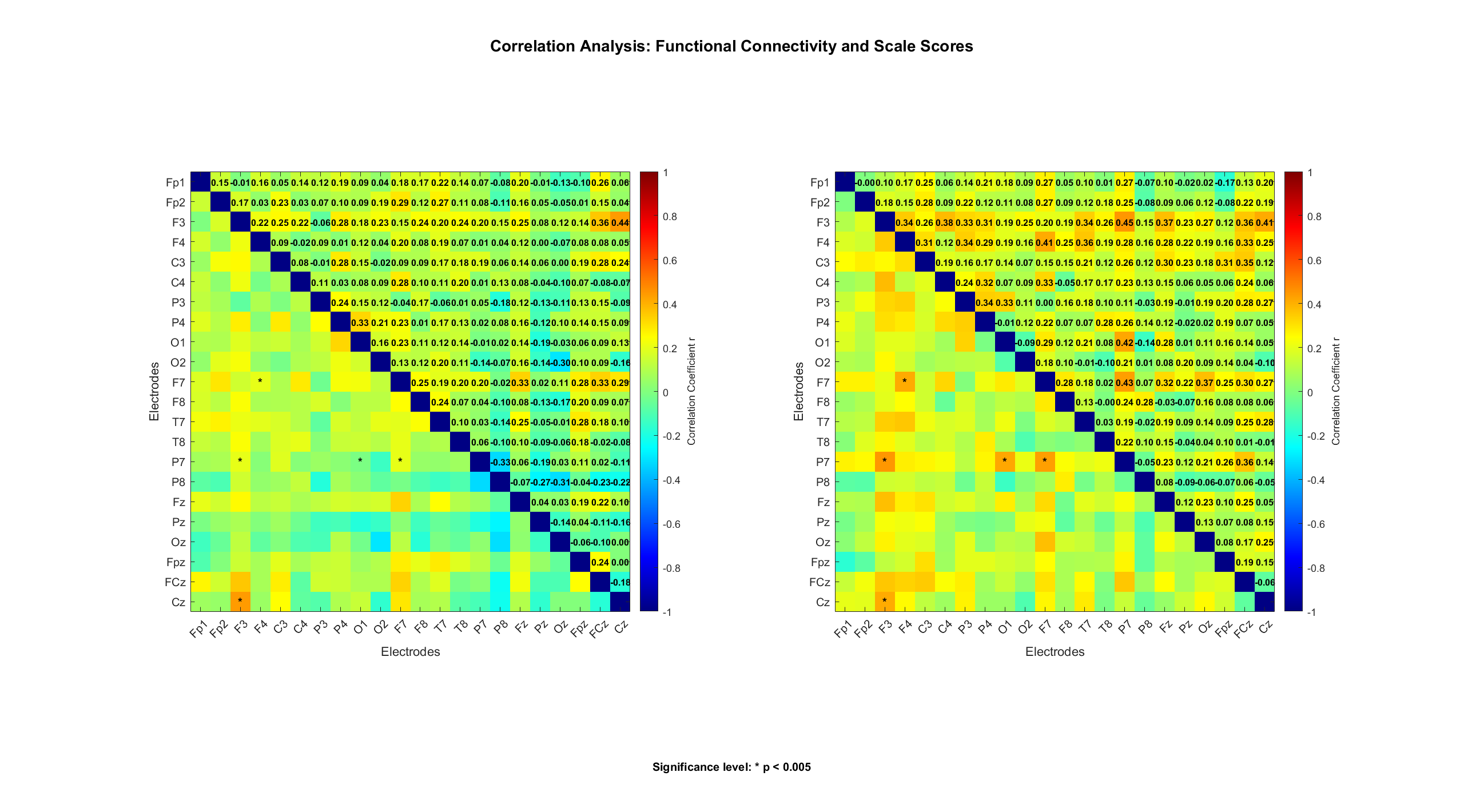


**Supplementary Figure 9. Correlation Analysis between Baseline EEG Functional Connectivity PLI in MDD Patients and Changes in Clinical Scales Following TECAS Treatment**

The baseline α-band PLI strength between parietal areas and frontal, occipital, and temporal areas (P4-F3, P4-F4, P4-C3, P4-P3, P7-P3, P8-P3, Oz-P3, Oz-P4, P8-P4) was significantly positively correlated with the change in HAMA-14 scores (average r = 0.456, *P* = 0.012). The values in the heatmap represent the correlation coefficient (r). The asterisks (*) below the heatmap indicate leads that are statistically significant after multiple comparison correction.


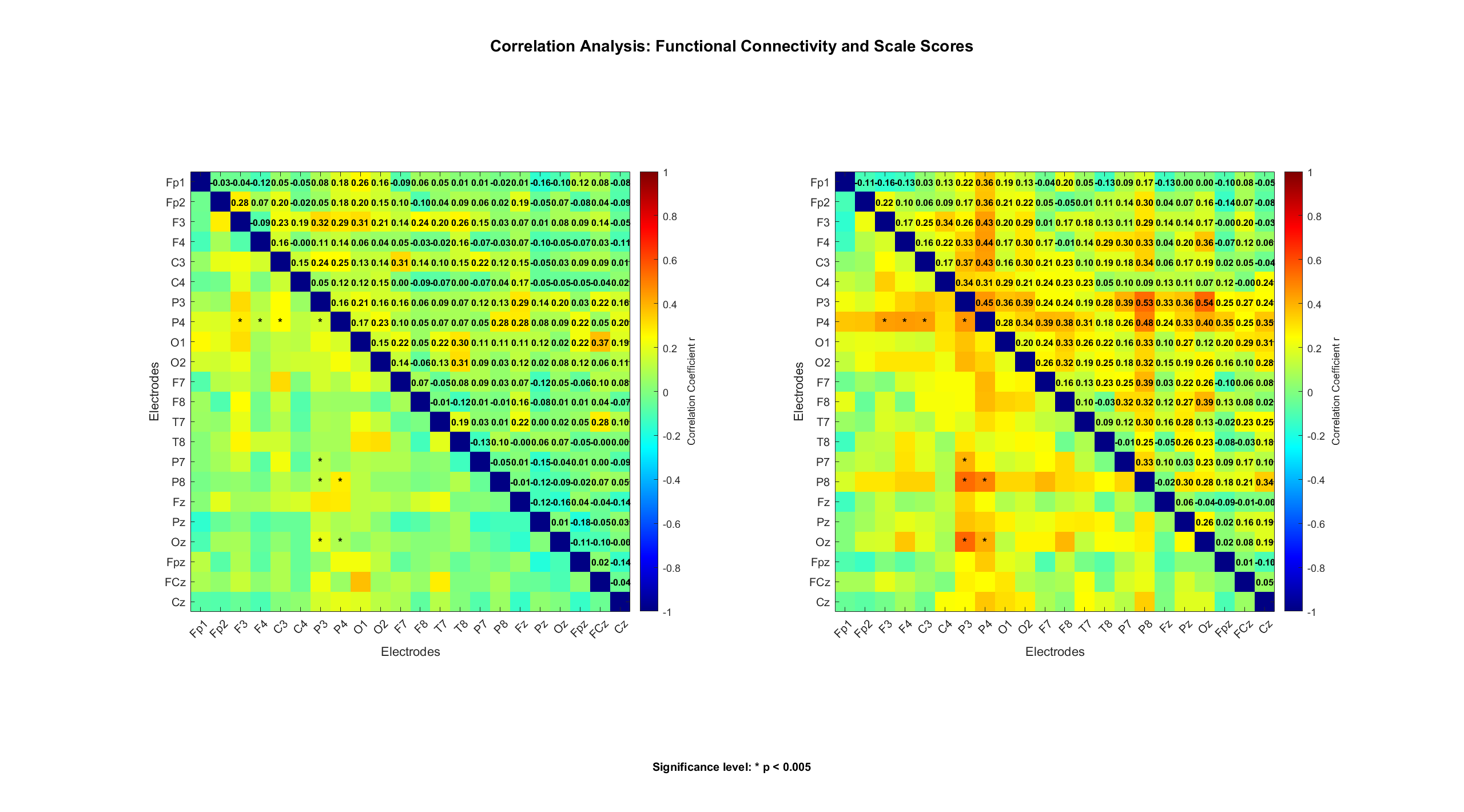


**Supplementary Figure 10. Correlation Analysis between Baseline EEG Functional Connectivity PLV in MDD Patients and Changes in Clinical Scales Following TECAS Treatment**

The baseline α-band PLV strength between parietal areas and frontal, occipital, and temporal areas (Cz-F3, P7-F3, F7-F4, F4-P3, P4-P3, FCz-P3, F7-P7, FCz-P7) was significantly positively correlated with the change in HAMA-14 scores (average r = 0.416, *P* = 0.016). The values in the heatmap represent the correlation coefficient (r). The asterisks (*) below the heatmap indicate leads that are statistically significant after multiple comparison correction.


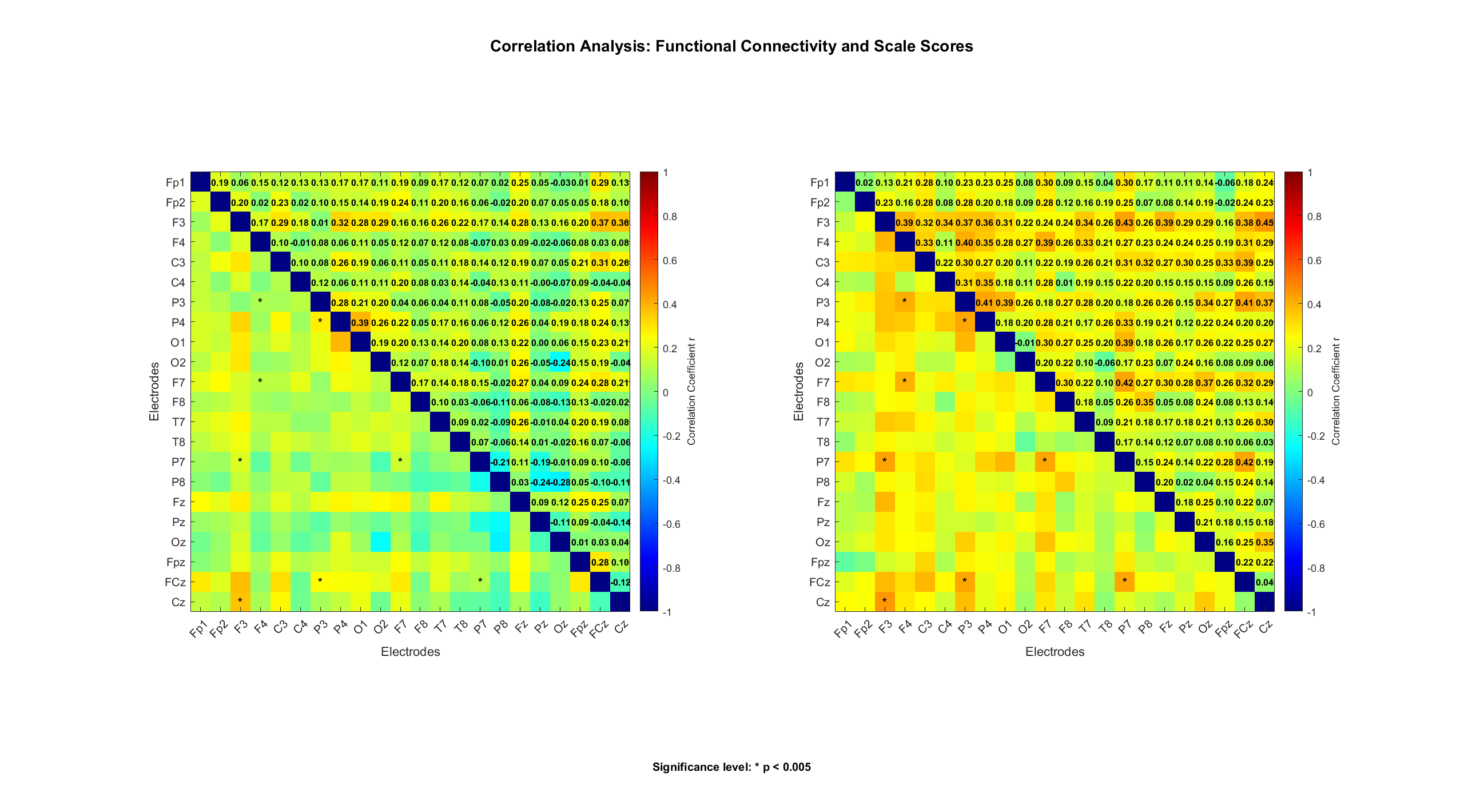


**Supplementary Figure 11. Correlation Analysis between Baseline EEG Functional Connectivity WPLI in MDD Patients and Changes in Clinical Scales Following TECAS Treatment**

The baseline α-band WPLI strength between parietal areas and frontal, occipital, and temporal areas (P4-F4, P4-C3, P4-P3, P8-P3, Oz-P3, P8-P4, Oz-P4) was significantly positively correlated with the change in HAMA-14 scores (average r = 0.442, *P* = 0.016). The values in the heatmap represent the correlation coefficient (r). The asterisks (*) below the heatmap indicate leads that are statistically significant after multiple comparison correction.


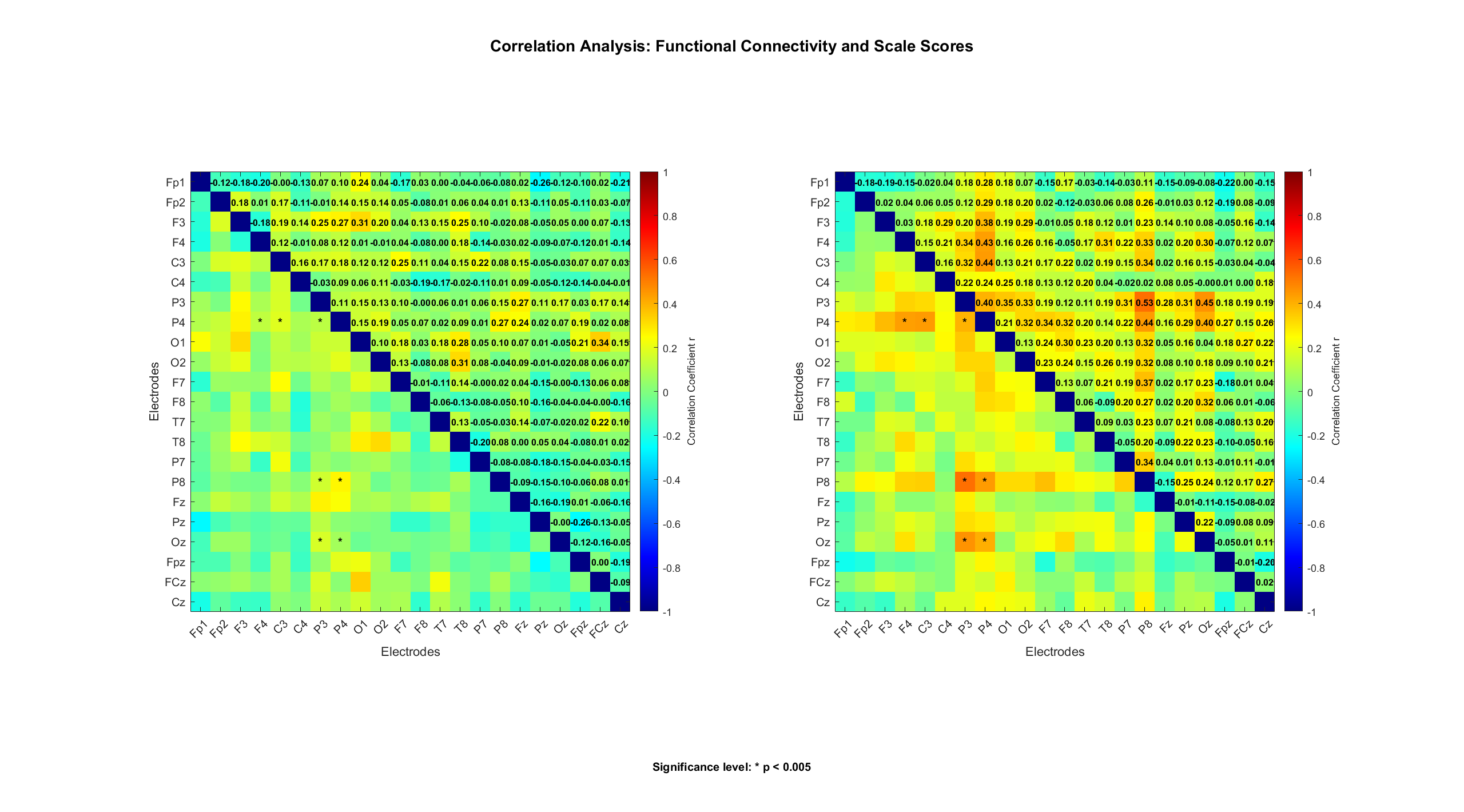


**Supplementary Figure 12. Correlation Analysis between Baseline EEG Functional Connectivity WPLI in MDD Patients and Changes in Clinical Scales Following TECAS Treatment**

The baseline γ-band WPLI strength between parietal areas and frontal, occipital, and temporal areas (FCz-F4, FCz-F8) was significantly positively correlated with the change in HAMA-14 scores (average r = 0.442, *P* = 0.016). The values in the heatmap represent the correlation coefficient (r). The asterisks (*) below the heatmap indicate leads that are statistically significant after multiple comparison correction.


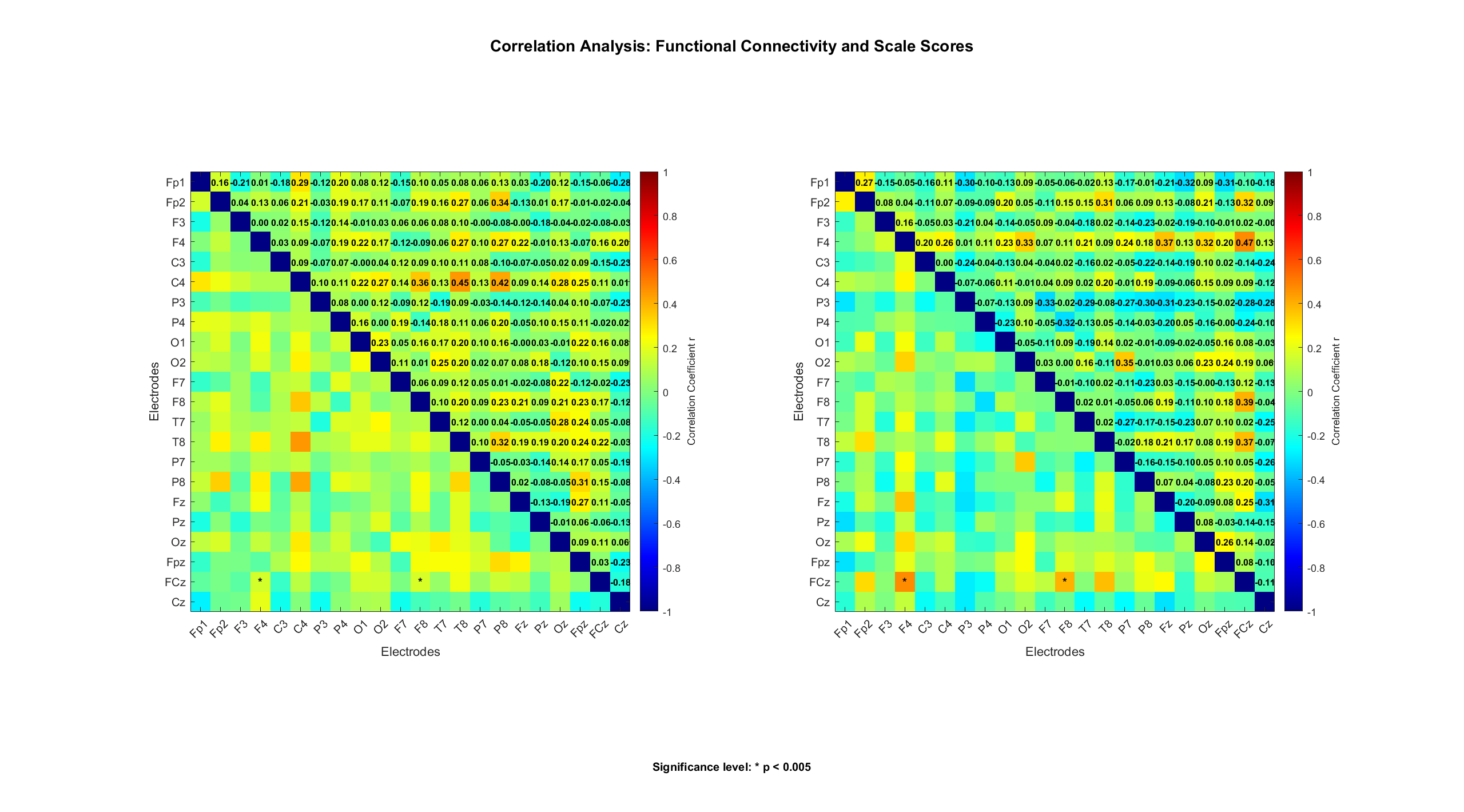


**Supplementary Figure 13. Correlation Analysis between Baseline EEG Functional Connectivity COH in MDD Patients and Changes in Clinical Scales Following TECAS Treatment**

The baseline α-band COH strength between the left temporal region (electrode P7) and the frontal, temporal, and parietal regions was significantly positively correlated with the change in HAMD-17 scores after TECAS treatment (average r = 0.434, *P* = 0.048). The values in the heatmap represent the correlation coefficient (r). The asterisks (*) below the heatmap indicate leads that are statistically significant after multiple comparison correction.


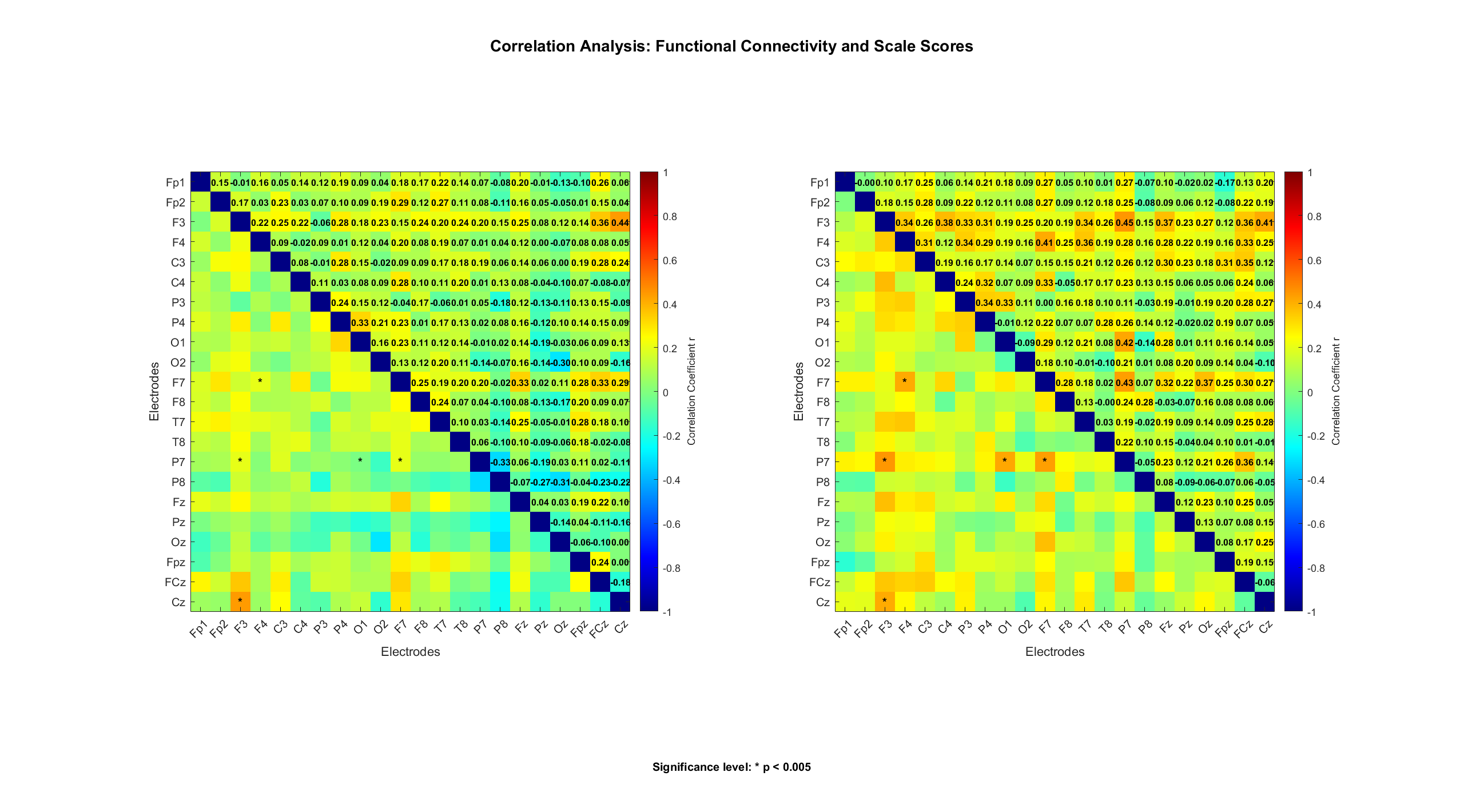


**Supplementary Table 1. Anxiety‑Adjusted Baseline Functional Connectivity (α-band COH metric) and Depression Reduction**

As shown below, the effect of baseline functional connectivity (α-band COH metric) on depression reduction was not significant except for the Cz-F3 electrode pair ($\text{F}_{\text{(1,47)}}$= 5.519, *P* = 0.023, *η*² = 0.105) after controlling for baseline anxiety (*P* > 0.05). The covariate effect of anxiety reduction was significant for all connections (all *P* < 0.05).

| Connection | Group Effect | | | | Anxiety Covariate Effect | | |
| --- | --- | --- | --- | --- | --- | --- | --- |
|  | Coefficient | *F* | *P* | Partial *η*² | Coefficient | *P* | Partial *η*² |
| P7-F3 | 0.2756 | 0.0017 | 0.9677 | <0.0001 | 0.4534 | 0.0063 | 0.1483 |
| P7-O1 | -10.1956 | 2.5745 | 0.1153 | 0.0519 | 0.5572 | 0.0006 | 0.2233 |
| P7-F7 | 0.8946 | 0.0216 | 0.8837 | 0.0005 | 0.4463 | 0.0066 | 0.1465 |
| Cz-F3 | 18.2835 | 5.5190 | 0.0231 | 0.1051 | 0.3152 | 0.0370 | 0.0893 |
| F7-F4 | 1.3953 | 0.0369 | 0.8485 | 0.0008 | 0.4440 | 0.0064 | 0.1479 |

**Supplementary Table 2. Anxiety‑Adjusted Baseline Functional Connectivity (δ-band COH metric) and Depression Reduction**

As shown below, the effect of baseline functional connectivity (δ-band COH metric) on depression reduction was not significant after controlling for baseline anxiety (*P* > 0.05). The covariate effect of anxiety reduction was significant (*P* < 0.01).

| Connection | Group Effect | | | | Anxiety Covariate Effect | | |
| --- | --- | --- | --- | --- | --- | --- | --- |
|  | Coefficient | *F* | *P* | Partial *η*² | Coefficient | *P* | Partial *η*² |
| Fz-C4 | -12.6598 | 1.5081 | 0.2255 | 0.0311 | 0.4028 | 0.0083 | 0.1393 |

**Supplementary Table 3. Anxiety‑Adjusted Baseline Functional Connectivity (β-band PLI metric) Associated with Treatment Response**

As shown below, the group effect was significant for all connections (all *P* < 0.01). The covariate effect of anxiety was not significant for any connection (*P* > 0.05) except for the O2-C4 electrode pair ($\text{F}_{\text{(1,47)}}$= 4.627, *P* = 0.037, *η*² = 0.090).

| Connection | Group Effect | | | | Anxiety Covariate Effect | | |
| --- | --- | --- | --- | --- | --- | --- | --- |
|  | Coefficient | *F* | *P* | Partial *η*² | Coefficient | *P* | Partial *η*² |
| O1-F3 | -0.0131 | 8.6459 | 0.0051 | 0.1554 | 0.0007 | 0.0835 | 0.0624 |
| Pz-F3 | -0.0117 | 10.0277 | 0.0027 | 0.1758 | 0.0004 | 0.2473 | 0.0284 |
| C4-F4 | -0.0162 | 7.6679 | 0.0080 | 0.1403 | 0.0005 | 0.3206 | 0.0210 |
| F7-F4 | -0.0120 | 7.9653 | 0.0070 | 0.1449 | -0.0001 | 0.8922 | 0.0004 |
| F8-F4 | -0.0171 | 11.0413 | 0.0017 | 0.1902 | 0.0005 | 0.2701 | 0.0258 |
| P7-F4 | -0.0157 | 14.1082 | 0.0005 | 0.2309 | 0.0004 | 0.3388 | 0.0195 |
| Pz-F4 | -0.0197 | 15.1111 | 0.0003 | 0.2433 | -0.0003 | 0.5221 | 0.0088 |
| O2-C4 | -0.0157 | 8.1573 | 0.0064 | 0.1479 | 0.0011 | 0.0366 | 0.0896 |
| Fz-T8 | -0.0115 | 7.2438 | 0.0098 | 0.1335 | 0.0002 | 0.5360 | 0.0082 |
| Cz-Fz | -0.0155 | 10.1713 | 0.0025 | 0.1779 | 0.0002 | 0.5805 | 0.0065 |
| Cz-Pz | -0.0150 | 9.9622 | 0.0028 | 0.1749 | -0.0001 | 0.8805 | 0.0005 |
| Cz-Fpz | -0.0112 | 8.8533 | 0.0046 | 0.1585 | 0.0002 | 0.6445 | 0.0046 |
| Cz-FCz | -0.0188 | 15.3509 | 0.0003 | 0.2462 | 0.0003 | 0.5482 | 0.0077 |

**Supplementary Table 4. Anxiety‑Adjusted Baseline Functional Connectivity (β-band WPLI metric) Associated with Treatment Response**

As shown below, the group effect was significant for all connections (all *P* < 0.01). The covariate effect of anxiety was not significant for any connection (all *P* > 0.05).

| Connection | Group Effect | | | | Anxiety Covariate Effect | | |
| --- | --- | --- | --- | --- | --- | --- | --- |
|  | Coefficient | *F* | *P* | Partial *η*² | Coefficient | *P* | Partial *η*² |
| C4-F4 | -0.0294 | 9.3048 | 0.0037 | 0.1653 | 0.0004 | 0.6177 | 0.0053 |
| F8-F4 | -0.0278 | 10.3977 | 0.0023 | 0.1812 | 0.0000 | 0.9576 | 0.0001 |
| Pz-F4 | -0.0280 | 8.3260 | 0.0059 | 0.1505 | -0.0007 | 0.4024 | 0.0150 |
| Cz-C3 | -0.0234 | 7.9524 | 0.0070 | 0.1447 | 0.0003 | 0.7034 | 0.0031 |
| FCz-C4 | -0.0221 | 10.4902 | 0.0022 | 0.1825 | 0.0007 | 0.2751 | 0.0253 |
| Cz-Fz | -0.0275 | 9.3390 | 0.0037 | 0.1658 | 0.0000 | 0.9543 | 0.0001 |
| Cz-FCz | -0.0251 | 7.9765 | 0.0069 | 0.1451 | 0.0005 | 0.5664 | 0.0070 |
